# Impact of Intrauterine Insults on Fetal and Postnatal Cerebellar Development in Humans and Rodents

**DOI:** 10.3390/cells13221911

**Published:** 2024-11-19

**Authors:** Judith A. W. Westerhuis, Jeroen Dudink, Bente E. C. A. Wijnands, Chris I. De Zeeuw, Cathrin B. Canto

**Affiliations:** 1 Netherlands Institute for Neuroscience, Royal Netherlands Academy of Arts and Sciences, 1105 BA Amsterdam, The Netherlands; judithwesterhuis@hotmail.com (J.A.W.W.); c.dezeeuw@erasmusmc.nl (C.I.D.Z.); 2 Department of Neonatology, Wilhelmina Children’s Hospital, University Medical Centre Utrecht, 3584 EA Utrecht, The Netherlands; j.dudink@umcutrecht.nl (J.D.); b.e.c.a.wijnands-2@umcutrecht.nl (B.E.C.A.W.); 3 Department of Neuroscience, Erasmus Medical Center, 3015 AA Rotterdam, The Netherlands

**Keywords:** cerebellum, development, intrauterine insults, alcohol drug abuse, nicotine, stress, sleep, malnutrition, motor memory, infection

## Abstract

Many children suffer from neurodevelopmental aberrations that have long-term effects. To understand the consequences of pathological processes during particular periods in neurodevelopment, one has to understand the differences in the developmental timelines of brain regions. The cerebellum is one of the first brain structures to differentiate during development but one of the last to achieve maturity. This relatively long period of development underscores its vulnerability to detrimental environmental exposures throughout gestation. Moreover, as postnatal functionality of the cerebellum is multifaceted, enveloping sensorimotor, cognitive, and emotional domains, prenatal disruptions in cerebellar development can result in a large variety of neurological and mental health disorders. Here, we review major intrauterine insults that affect cerebellar development in both humans and rodents, ranging from abuse of toxic chemical agents, such as alcohol, nicotine, cannabis, and opioids, to stress, malnutrition, and infections. Understanding these pathological mechanisms in the context of the different stages of cerebellar development in humans and rodents can help us to identify critical and vulnerable periods and thereby prevent the risk of associated prenatal and early postnatal damage that can lead to lifelong neurological and cognitive disabilities. The aim of the review is to raise awareness and to provide information for obstetricians and other healthcare professionals to eventually design strategies for preventing or rescuing related neurodevelopmental disorders.

## 1. Introduction

Neurodevelopmental disorders, characterized by cognitive, neurological, and psychiatric complications, affect one in six children in industrialized nations, causing enduring consequences with extensive societal and economic ramifications [1]. Detrimental prenatal environments can modify brain development, potentially leading to neurodevelopmental disorders [2,3]. The intricate influence of environmental variables on human fetal neurodevelopmental conditions is yet to be precisely determined. The developmental timelines of brain regions vary, indicating that the type, severity, and timing of harmful exposures are crucial in determining resulting aberrations [4].

The cerebellar anlage appears in humans from embryonic day (E) 29 and in rodents from E8.5 onwards [5,6,7]. It grows rapidly during gestation and continues to mature until late postnatal life [8] with long-range connections from the cerebellum to the thalamus and cerebrum starting to develop prenatally and continuing to develop postnatally.

Within the cerebellum, there are regional differences in the development of the anatomical local and long-range connections [9,10]. The differential spatiotemporal cerebellar development is causally related to a distinctive functional development. Shifts in neuroscience perspectives have reframed the cerebellum, traditionally overshadowed by the corticocentric viewpoint, acknowledging its role in both motor and non-motor functions with distinct cerebellar areas processing diverse functions [11,12,13,14]. Current understandings affirm the cerebellum’s pivotal role in modulating motor control, precision, and learning, correlating cerebellar damages with substantial motor disorders [15], but Schmahmann’s elucidation of the “Cerebellar Cognitive Affective Syndrome” marked a paradigmatic shift, extending cerebellar functionality to encompass cognitive and affective domains [16]. As did the work of Andreasen and colleagues, who developed a model that postulates the emergence of ‘cognitive dysmetria’ with ‘poor mental coordination’ in case of disruption of the functional connectivity between prefrontal regions, the thalamic nuclei, and the cerebellum [17,18]. Contemporary research corroborates the cerebellum’s integration in attention, behavior, cognition, and language but also associates its aberrations with developmental anomalies like dyslexia, autism, and attention deficit hyperactive disorder [12,13,19].

All this underlines the cerebellum’s vulnerability to detrimental environmental exposures happening during different time points of gestation [20], highlighting the significance of studying the associated biomedical mechanisms and consequences underlying the diverse set of cerebellar neurodevelopmental problems.

## 2. Scope

The development of the cerebellum has been a focus of many outstanding lines of research, and, over the years, multiple reviews have been published describing the development of the cerebellum and its cyto-architecture (for example [8,13,21,22,23]). Throughout prenatal development, the cerebellum can be affected by both genetic and intrauterine insults [20], often leading to poor neurodevelopmental outcomes. We are focusing our review on maternal insults during pregnancy that are known to affect a myriad of human mothers; these include maternal substance abuse, intrauterine stress-related events, undernutrition, and infection, such as chorioamnionitis (CA). The aim of this review is to summarize, compare, and align human and rodent research such that we obtain a better understanding of the impacts of intrauterine insults on fetal and postnatal cerebellar development. With this, we want to raise awareness and provide information for obstetricians and other healthcare professionals to design strategies for preventing or rescuing related neurodevelopmental disorders.

## 3. Cerebellar Anatomy

**Figure 1:**
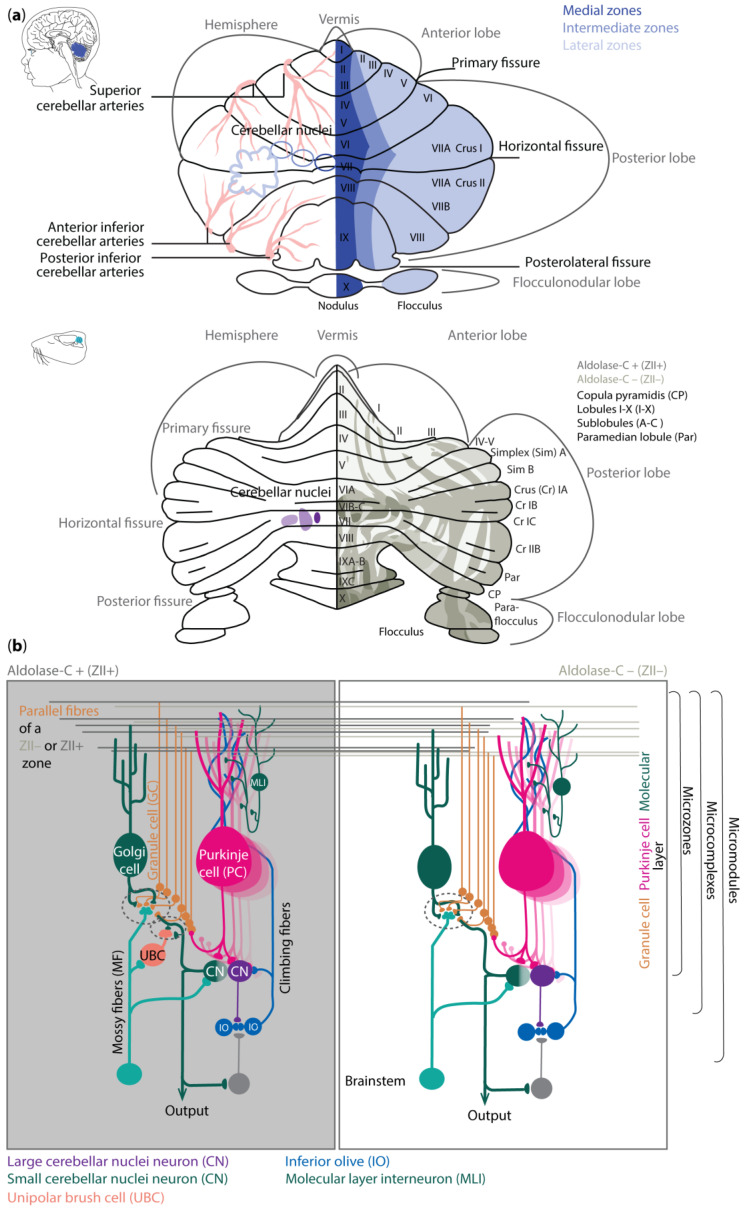
Anatomy of the human and rodent cerebellum. (**a**) An unfolded view of the human (**top**) and rodent (**bottom**) cerebellum. (**Top**) The three cerebellar zones (right hemisphere: medial, intermediate, and lateral) are interconnected with different parts of the cerebellar nuclei (left hemisphere), indicated with the same color-coding. The areas of the cortex served by the cerebellar arteries are indicated on the left (adapted from [29]). (**Bottom**) Schematic of the mouse cerebellum with sagittal microzones visualized based on the expression levels of aldolase-C (also known as zebrin II (ZII); right hemisphere). Different shades of gray indicate how strong (dark gray = strong ZII+, white = ZII−) aldolase-C is expressed. (**b**) The cytoarchitectures of neighboring microzones, such as those indicated in (**a**, **bottom**), are almost identical (adapted with permission from [30]). The microzones, microcomplexes, and micromodules are all built up in a comparable manner. Individual zones communicate with each other through, e.g., parallel fibers of granule cells that cross the zones/complexes/modules.

Regional differences in cerebellar vulnerability are underlined by anatomical differences. The cerebellar hemispheres can be similar to the vermis, divided into individual lobules I–X (reviewed in [24]), which are split into the anterior (lobule I–V), posterior (VI–X), and flocculonodular lobe by fissures (Figure 1a). The anterior lobe has been suggested to be mainly involved in sensorimotor control of limb movements, whereas the posterior lobe including for example Crus 1 and 2 may be more engaged in facilitating cognitive processes [25,26,27,28]. The flocculonodular lobe on the other hand forms the phylogenetically older vestibulocerebellum, controlling balance and compensatory eye movements [9,24].

The cerebellum receives information via the three-layered and folded cerebellar cortex that surrounds the cerebellar nuclei (CNs) (Figure 1b). The sole output neurons of the cerebellar cortex are inhibitory Purkinje cells (PCs). They are located in the middle layer, also referred to as the PC layer (PCL). The expression patterns of different genes, such as *aldolase-C* (*Zebrin II/ZII*) [31], small heat shock protein *(HSP)25* [32], *GRID2* [33], and *phospholipase Cbeta4* (*PLCB4*) [34], reveal a pattern of parasagittal stripes of PCs, around which the rest of the cerebellar cortex is organized [35]. For example, the climbing fiber afferents (CFs) from specific subnuclei of the inferior olive to the microzones of PCs as well as the PC efferents to the various cerebellar nuclei adhere to this topographical organization. The occurrence of parasagittal zones is relatively well preserved across species, finding its foundation already in early embryology [36]. As a result, mutations in those genes can lead to severe neurodevelopmental issues [37,38,39]. 

Despite the general organization of parasagittal zones that can be identified with molecular and anatomical tools, mapping the molecular, and spatial composition of the cerebellar cortex has also revealed differences among mammalian species [40,41,42]. For example, in human, one can distinguish transient cell types and cytoarchitectures that cannot be identified with the same approach in mice [40]. Likewise, there are specific cell subtypes of PCs and molecular layer interneurons (MLIs), as well as region-specific gene expression profiles, that can be found in the cerebellum of primates but not in that of mice [42]. Moreover, in humans and primates, region-selective gene expression has predominantly been observed in granule cells (GCs), whereas, in rodents, the region-selective gene expression has mainly been shown in PCs [41,42]. 

The differences in expression patterns of cerebellar neurons in the various species are likely to have functional implications in terms of electrophysiological properties and local network connections. In rodents, ZII+ PCs can be associated with relatively low baseline simple spike firing and upbound changes following learning, whereas ZII− zone learning may be linked to relatively high baseline firing and downbound changes [30]. The foundation for such differences in learning profiles probably relies more on the expression profile of PC genes rather than between their synaptic inputs [30,43,44,45,46,47,48]. The modulation of simple spike firing of PCs is tuned by various inputs. Most of these inputs reside in the outermost layer of the cerebellar cortex, the so-called molecular layer (ML). The interneurons in this layer, the MLIs, have historically been divided into stellate and basket neurons, which inhibit PCs predominantly at their dendrites and soma, respectively. However, modern transcriptomics studies have revealed there is also a specific type of MLI that mainly inhibits other MLIs and disinhibits PCs [49]. PCs receive their excitatory inputs from CFs and parallel fibers (PFs). Whereas the CFs arise in the inferior olive, PFs are the axons of GCs, which are located in the GC layer (GL) where the mossy fibers (MFs) terminate. The GL also comprises inhibitory Golgi cells and, in the zebrin-positive microzones, excitatory unipolar brush cells (UBCs) [50]. All neurons in the GL increase the diversity of the MF information, which then ultimately is relayed via the PFs to the PCs. The PC axons project onto glutamatergic CN neurons that control the premotor and non-motor nuclei in the brainstem and diencephalon, as well as onto local interneurons and the gamma-aminobutyric acid (GABA)ergic projection neurons that provide the main feedback to the inferior olive [51,52]. It remains to be seen to what extent the PCs and CNs in humans and primates show the same intrinsic electrophysiological properties and default changes during learning as those found for mice. The finding that the ratio between the various neuronal subtypes differs among these species [53] and that the level of heterogeneity is more prominent in higher species [40,41,42], may point towards an enrichment in learning rules in primates. 

## 4. Embryology of the Cerebellum

**Figure 2:**
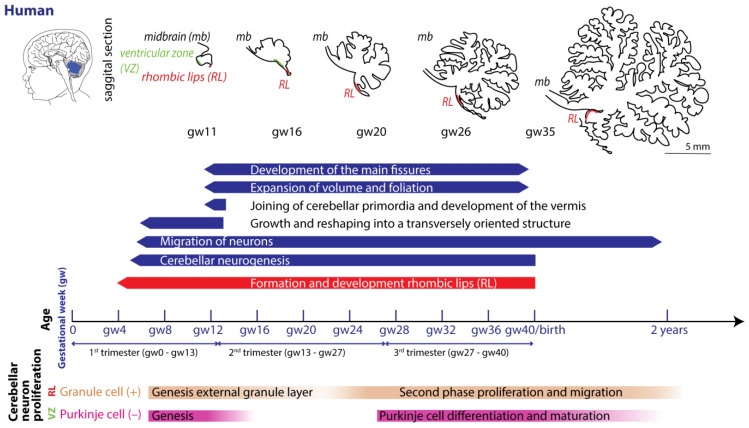
Developmental timeline of the human cerebellum. Top row: Sagittal sections of the outline of the human cerebellum at five timepoints during development, starting from gestational week (gw) 11 (adapted from [20]) with different aspects of the development being highlighted below. Bottom row: The timeline of the development of individual cerebellar cell types. The duration and timing of development is indicated with a color-coded bar with a distinct bar length. Excitatory (+) granule cells (orange) derive from the rhombic lips (RLs, red). Inhibitory (−) GABAergic Purkinje cells (pink) derive from the ventricular zone (VZ, green).

Each species exhibits particularities regarding gestation. Term parturition in women occurs around 280 days after the onset of their last menstrual period (Figure 2) [54].

**Figure 3:**
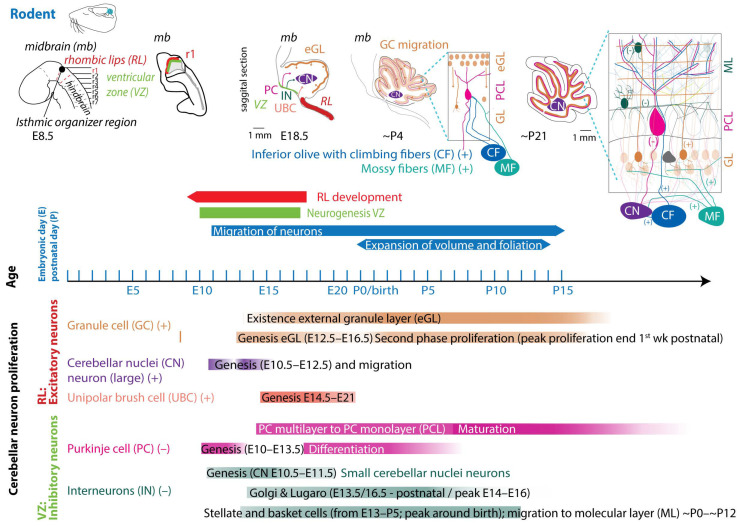
Developmental timeline of the rodent cerebellum. **Top row**: The first two outlines show the differentiation of the hind- and midbrain (mb) and the relative location of the cerebellar anlage [70]. Embryonic day (E)18.5 and postnatal ~(P)4 and P21 show the outlines of cerebellar midsagittal cuts (adapted from [71]). Arrowheads indicate the migration direction of individual neuron types. At ~P4 and P21 also, a zoom-in of the cerebellar cortex is presented to show the location of individual cell types in the (external) granule layer ((e)GL), Purkinje cell layer (PCL), and molecular layer (ML). The inputs of the two precerebellar systems, the climbing fibers (CFs) and mossy fibers (MFs), and the output to the cerebellar nuclei (CN) are also indicated. **Bottom**, the timeline of the development of the cerebellum and the timeline of the development of individual cerebellar cell types are drawn.

In mice and rats, gestation is shorter, lasting between 20 and 22 days (described in [55]). Regarding the comparison between cerebellar brain development in rodents and humans, one must consider different aspects, since they do not run proportionally in parallel [22,56]. The developmental journey of the human cerebellum commences 29/30 days after conception [5,6,7] and ends around 2–3 year after birth (Figure 2). The rodent cerebellum starts to develop around E8.5 and ends around postnatal week 3 (Figure 3) [5,6,7]. There are two progenitor zones, the ventricular zone (VZ) and the rhombic lips (RLs), at the start of cerebellar development. Forty to forty-five days after conception, the human cerebellar VZ splits into a VZ and a subventricular zone [57]. The RLs expand spatiotemporally and promote growth and maintenance of the posterior lobe. In mice, the VZ does not split, and RLs’ presence is transient [22]. The external granule layer (eGL) is a third progenitor zone later during development in both humans and rodents. Cerebellar maturation can be distinguished by a multifaceted, symbiotic cascade, encompassing gene expressions, electrical network interactions, and environmental factors, which are inherently interlinked with the evolution of the hindbrain (hb) and midbrain (mb). The transcription factors Otx2 and Gbx2, which are expressed anterior and posterior from the so-called isthmic organizer region between the mb and hb, are important for the general development of most of the rostral and caudal regions of the central nervous system [58]. Originating from the caudal-most primary neural tube vesicles, the rhombencephalon (hb) bifurcates into the metencephalon and myelencephalon. Segmented along the rostral–caudal axis into seven rhombomeres, the cerebellum’s emergence is facilitated by transcription factors and signaling molecules, leading to the formation of specialized epithelium, RL1, from the dorsal portions of the metencephalon [59] through bilateral expansion of the Alar plate [60]. The latter occurs in the presence of *Gbx2* and absence of *Otx2* and *Hoxa2* [58,61,62]. *Wnt* family members, fibroblast growth factors (especially *Fgf8* and *Fgf17*), *En1-2*, *Lmx-1*, and sonic-hedgehog (*shh*) play a major role in defining the isthmic organizer region and the anterior–posterior as well as the dorso-ventral patterning of cerebellar development [63,64,65]. Mutations in any of these genes lead to severe implications for cerebellar development or even death in rodents [66,67,68,69]. In rodents, around E9, cerebellar histogenesis starts. Just above the fourth ventricle, the two germinative compartments of the RLs are formed, adjacent to the roof plate and the VZ placed in the inner side. The bulges grow and give rise to the unitary cerebellar plate comprising the vermis and hemispheres. The cerebellar medial regions expand, an orthogonal rotation happens, and the cerebellar wing-like anlagen transform into a homogeneous cylindric vermis at E15.5. Cerebellar size expansion and increased lobular complexity occur from gestational week (gw) 20 in humans and from birth (postnatal day 0 (P0)) in mice.

### 4.1. Neurogenesis

In both humans and rodents, the RLs form the origin for all glutamatergic cerebellar neurons, whereas all GABAergic neurons, as well as glia cells, oligodendrocytes, and astrocytes, originate from the VZ (Figure 2 and Figure 3) [22,23,57]. Birth-dating studies showed that the projection neurons are produced first at the onset of cerebellar neurogenesis.

#### 4.1.1. Glutamatergic Neuron Development

In rodents, the RLs are defined by the expression of the mouse homolog of Drosophila atonal (ATOH1) transcription factor [72], forming the origin of all glutamatergic cerebellar neurons [73]. ATOH1 expression begins at E9.5 in mice, and from E10.5 to E12.5, progenitors leaving the rostral RLs give rise to the large CN neurons migrating to the surface of the cerebellar anlage, where they aggregate in the nuclear transitory zone. From there, they move inward from the PC plate to form the four CNs on both sides [74]. Progenitors migrating between E14 and E21 give rise to UBCs in distinct cerebellar areas [75]. Then, GCs start proliferating in response to shh signaling from PCs [76]. The GC lineage arises already around E8.75. GC progenitors exit the upper RLs, moving tangentially along the cerebellar surface, eventually covering the entire cerebellar anlage by E16 [77]. At least three transverse GC progenitor zones are identified by gene expression and birth-dating. Postmitotic GCs migrate from eGL to the inner GL guided by the Bergmann glia fibers, which are oriented in the same plane (Figure 3 top). Thereby, the eGL topography is projected into the nascent inner GL [77]. After birth, the GL is an eight-layered structure with another layer of proliferating granule precursor cells [77,78]. The proliferation window of murine GCs progenitors closes only at the end of the second postnatal week in rodents, when the last postmitotic GCs, from the deepest portion of the eGL, migrate inwardly to the nascent GL, marking the end of the eGL and ceasing ATOH1 expression [72,79]. Interestingly, cell-fate specification among the cerebellar VZ and RL is not absolute. A so-called posterior transitory zone expresses genes to develop bipotent progenitors for cerebellar glutamatergic neurons [80].

#### 4.1.2. GABAergic Neuron Development

The VZ is defined by the pancreas transcription factor 1-a (Ptf1a), giving rise to GABAergic neurons. A Ptf1a-neurogenin 1/2 (Neurog1/2)- early B-cell factor 2 (EBF2) regulatory network is implicated in PC subtype specification [81]. In rodents, PCs are born between E10 and E13 and undergo terminal mitosis. Dividing VZ precursors emigrate into the cerebellar prospective white matter via the cerebellar plate and form an array of clusters (E14–E18), which are suggested to aggregate into microzones, also called topographical organization centers (TOCs) (Figure 1). These TOCs are not only specific for ingrowing afferent precerebellar MF and CF inputs, as well as interneurons, but also for subsets of glia cells and migrating GCs [82]. As the PC clusters disperse into parasagittal stripes (Figure 1), all components disperse with them, forming the adult cerebellar parasagittal architecture. MFs disconnect and form local connections with GCs within the zone. At least five molecularly distinct PC subgroups have been identified throughout development with distinctive levels of Foxp1 and Foxp2, respectively. Foxp1+/Foxp2+ PCs strongly express reelin receptors and lack Ebf2. Reelin controls PC migration [83,84,85,86]. Early-born PCs are likely to become ZII+ during adulthood, while late-born PCs adopt the ZII− phenotype, which is in line with the high aldolase-C expression in the phylogenetically older vestibulocerebellum (Figure 1a) [87]. The postnatal development of PCs can be divided into different stages. The first distinction can be made between intrinsic maturation by PCs themselves and guided maturation by stimulation of other cell types, such as GCs [88]. In rodents, intrinsic growing starts with a fast somatic growth from P0–P9 followed by a rapid dendritic growth from P9–P18 [89,90], with more processes growing outwards from the soma. During the second postnatal week, the processes become more complex by growing rapidly and increasing the number of branches. This is the start of the dendritic tree, which will be completed around postnatal week 4 in rodents [91]. CFs are also involved in the dendritic arborization of PCs during these stages, presumably by stimulating PCs. This may explain why higher-order mammals, including humans, show both a higher level of persistent multiple CF innervation and a higher complexity of the dendritic trees of their PCs [92]. GABAergic interneurons in the CN are born between E10.5 and E11.5, while Golgi cells are born at approximately E13.5–postnatally (peak around E14–E16) [6,23,93,94]. Late-born GABAergic interneurons, including stellate and basket cells, derive from secondary precursors in the prospective white matter at later stages (from E13 to P5 with a peak around birth) [95]. Thus, cerebellar neuronal subtypes depend on when and where they are generated from neural progenitors. Additionally, the cerebellum accommodates astrocytes, glia, and oligodendrocytes, the origins of which are not fully discovered [96]. The start and end time as well as the total duration of the developmental timeline of cerebellar cell types display distinct susceptibilities to environmental insults and genetic mutations [97].

### 4.2. Embryology of the Precerebellar System

Interior olive neurons are derived from the dorsal neuroepithelium, the caudal hb (RL 6–8) at E9.5 to E11.5 in rodents. Olivocerebellar projections, the CFs, are being formed at E17.5 [98], and at the late embryonic stage, the olivocerebellar bundle already shows an organized topographic projection pattern. Neurons in a particular subnucleus of the inferior olive project via their CFs to a specific part of the cerebellum. The bundles with CFs run contralaterally through the inferior cerebellar peduncle. Axons that leave the peduncle rostrally project to the vermis, whereas CFs innervating the other cerebellar areas pass through the more caudal parts of the inferior cerebellar peduncle [99]. During early development, CF axons form a plexus with abundant branching, while in the second postnatal week, axonal branches disappear, and those so-called nest terminals grow into an entire CF terminal in the following weeks. The latter form the distinct one-to-one synaptic connection in rodents (Figure 3, for comparison in human, see [92]). MFs are derived from the dorsal neuroepithelium 1 domain of the caudal hb (RL 6–8) and are generated at slightly later stages (E10.5–E16.5) compared to CFs [100]. Initially, axonal fibers of GCs that receive MF input form contacts with the soma of PCs, and only around P5–P15 do they start to turn into the typical parallel fibers that establish synaptic contacts with the dendrites of PCs [91].

## 5. Extrinsic Deterrents Influencing Cerebellar Development

As mentioned in the Scope Section, multiple genetic and external conditions can significantly hinder both pre- and postnatal development of the cerebellum, resulting in impaired maturation and functionality. In this review, we focus on main common maternal disruptors during prenatal stages, including (1.) teratogenic exposures during pregnancy to substances like alcohol, nicotine, cannabis, and opioids; (2.) increases in cortisol level (stress); (3.) intrauterine growth restriction (IUGR) resulting from, e.g., malnutrition; and (4.) chorioamnionitis (CA) [2,20].

### 5.1. Maternal Substance (Ab)use and Cerebellar Maturation

The cerebellum has been suggested to be sensitive to drugs with abuse liability, including alcohol, nicotine, cannabis, and opioids, leading to decreased cerebellar volume, increased apoptosis, and behavioral differences [101,102,103,104,105,106,107]. The critical periods of sensitivity to drugs of abuse coincide with the long cerebellar neuron proliferation and migration phase, which coincides with the embryonic and postnatal first three weeks of a rodents life and the third trimester of gestation until 1.5 years in humans [107,108]. It has been suggested that drugs act by mimicking or interfering with normal endogenous neurotransmitter–receptor interactions and thereby misleading the endogenous timing and sequence of endogenous neurotransmitter–receptor interactions.

#### 5.1.1. Maternal Alcohol Consumption—Impact on Cerebellar Maturation

One of the primary challenges in studying the effects of maternal alcohol consumption on fetal cerebellar maturation in humans is the inherent limitations of fetal neuroimaging. These include a low signal-to-noise ratio in ultrasound imaging, motion artifacts in MRI scans, limited availability of postmortem data, and the absence of experimental human studies. Despite these obstacles, research in this area is important to understand the impact of alcohol exposure on cerebellar development.

Excessive alcohol exposure during pregnancy results in results with fetal alcohol spectrum disorder (FASD), which usually comes with symptoms such as balance disturbances and impairment of motor skills [109,110]. Cerebellar degeneration might contribute to some of the cognitive disabilities that can be observed in children with FASD [110]. As the cerebellum undergoes rapid growth during the second and third trimesters of pregnancy [111], it is particularly vulnerable to alcohol exposure during this critical period [112].

Studies reveal that early cessation of alcohol use upon pregnancy recognition can mitigate some adverse outcomes, as no significant differences in fetal growth measures are observed between those who abstained and the non-exposed groups [113]. However, heavy alcohol consumption that continues after conception is associated with altered fetal cerebellar development, showing a reduced transcerebellar diameter [113]. Fetal neuroimaging findings on the cerebellar vermis measurements are mixed, with some studies showing decreased vermis lobules I–V sizes in children exposed to alcohol in utero, while others found no significant differences [114,115,116,117]. Inconsistent results are also noted in the literature regarding the impact of prenatal alcohol exposure on overall cerebellar development [118]. While some studies highlight smaller cerebellar volumes in alcohol-exposed groups [113], others report no significant reductions in cerebellar growth [2].

Furthermore, prenatal alcohol exposure has been linked to changes in cerebellar white matter integrity, potentially contributing to impaired motor and cognitive functions [119,120]. These neurodevelopmental disruptions are thought to arise from altered myelination [119] and impaired neural transmission in cerebellar tracts [121]. fMRI analysis also suggests distinct patterns of network activation regarding working memory in two differently alcohol-exposed groups, revealing dose sensitivity for alcohol [122]. Despite these findings, variability in outcomes across studies underscores the complex interplay of alcohol exposure patterns, individual differences, and methodological challenges in assessing the effects of alcohol on fetal brain development. 

In rodents, prenatal maternal ethanol administration limited to E8 or E9 leads to differences in cerebellar shape and decreased volume at E17 [123,124]. Although volume changes are not visible anymore, shape differences together with behavioral changes still exist in adult mice [125,126]. Ethanol administration at E7 does not alter cerebellar volumes at E17 [127]. The latter does not come as a major surprise, as the development of the cerebellum starts only at E8.5. Ethanol exposure for the first gw results in a decreased proportion of GABAa receptors (subunit α1) in the adult cerebellum [128]. Alcohol administration during almost the whole pregnancy results in increased oxidative stress and apoptosis markers, as well as decreased GLUR1, PSD95 and ILK expression [129]. However, in another study, neither the amount of PCs nor the cerebellar-to-body-weight ratio reduces at P10 when ethanol is administered during the third week of pregnancy [130], indicating that ethanol exposure has a higher impact on PCs neurogenesis than on PC differentiation. Yet, another study revealed that alcohol exposure during both E12–19 and P2–9 resulted in decreased numbers of inhibitory interneurons in lobule II, lower numbers of PCs in lobules II, IV–V, and IX and decreased volumes of lobules II, IV–V, VI–VII, IX, and X of the vermis at P16 [131]. Ethanol treatment before and during pregnancy as well as during lactation leads to morphological differences in the eGL, GCs, and Bergmann glia in offspring [132]. Whether the morphological differences are caused by the maternal or prenatal ethanol exposure or the combination of both remains unclear.

Postnatally, a ‘temporal window of vulnerability’ of the cerebellum for harmful effects of alcohol administration has been described [133]. This window of vulnerability starts with the birth of the pups and ends with the start of the second postnatal week [133,134,135], a timeframe roughly corresponding to the beginning of the third trimester in humans. Alcohol administration for only 1 day during the first postnatal week results in reduced brain weight at P10, with the cerebellum being diminished the most [101]. The biggest loss of PCs after ethanol treatment occurs after ethanol consumption around P4–6, although lobule-specific sensitivity differences exist [135]. More specifically, alcohol administration between P2–P5 leads to reduced volumes of the GL and ML and a significant loss of PCs and GCs in all lobules of the vermis, except for the PCs of lobules VI and VII [101,134,136,137,138,139]. The ethanol sensitivity of PCs in lobules I–V and IX decreases over time, whereas lobule VII’s vulnerability increases towards the end of the first postnatal week [135]. Also, the numbers of MLIs decreases after ethanol exposure at P4–6. Nevertheless, the amount of spontaneous inhibitory synaptic currents as well as the hyperpolarization-activated inward current (Ih) amplitudes in the remaining PCs increase [140]. Regarding external inputs, the CF distribution is altered as the CF terminals reduce in volume and immunostaining intensifies relative to the PC volumes in P14 rats [141]. There is also a reduction in CF terminal volumes relative to PC volumes in P40 rats [142], suggesting decreased numbers of CF-to-PC connections. Ethanol exposure during both the first and second postnatal week, specifically P4–9, results in reduced overall cerebellar weight, reduced volumes of lobules I–IV and IX–X, reduced numbers of GCs and PCs, increased microglia density, and activated microglia [130,136,143,144]. Whether microglia are still affected by ethanol exposure later in life remains unclear. While Gursky et al. (2020) found increased microglia densities, Cealie and colleagues did not find significantly affected microglia morphology, density, or microglia–PC interaction in lobule IV/V using in vivo two-photon imaging and fixed tissue [145,146]. Minor changes were found in the ML and PCL.

The literature is inconsistent regarding the effects of alcohol exposure after the ‘window of vulnerability’ after the first postnatal week. No alterations in cerebellar weight, amount of PCs and GCs, or area loss in the GL or ML were found after alcohol exposure between P7 and P13 in most literature [133,134,135,141,142]. Hence, three studies show effects of alcohol administration after P7. Two studies show CF distributional differences after ethanol exposure between P7–9 [141,142] and one study shows that ethanol administration during P10–12 can result in a thinner eGL and reduced numbers of GCs in the GL and a reduced size of the ML [147].

There remain several open questions regarding which factors contribute to this window of vulnerability to ethanol exposure and to what extent differential gene expression plays a role in that. The expression of some neurodevelopmental genes varies between the first and second postnatal weeks, and ethanol-induced alterations have been shown for those genes [134]. The genes involved in cerebellar vulnerability to ethanol are the *nNOS* gene and the *CREB* gene. mRNA expression of the *CREB* gene increases at P4 compared to P10 after ethanol exposure [134]. PCs are also much more sensitive to ethanol exposure at P4–9 when CREB is not expressed in PCs [143], suggesting that under control conditions, the cAMP pathway plays a protective role in neonatal alcohol exposure during the first postnatal week. Mice with a mutation in the *nNOS* gene are more vulnerable to ethanol-induced behavioral changes and PC and GC losses [148].

Another pathway affected by ethanol exposure is the Wnt-signaling pathway. As mentioned above, Wnt is important during cerebellar maturation and development. After maternal ethanol exposure from E6 until birth, 3 of the 84 genes related to the Wnt pathway are down-regulated at P10, and 33 genes are down-regulated at P35 [149]. More specifically, at P20, *Wnt5a*, *Fed 6*, *Didxc*, *Axis 2*, *Pzd4*, *Fzd6*, and *EP300* gene expressions are decreased, and *Wnt5b* gene expression is increased [149,150]. Moreover, other pathways affected by maternal ethanol exposure are insulin/IFG-1 and Notch-signaling pathways at P30 [150].

Postnatally, ethanol exposure also effects gene regulation. A recent study looking at alcohol-induced transcriptomatic changes found 2440 dysregulated genes one day after ethanol exposure at P4 and 1348 dysregulated genes one day after ethanol exposure at P4–5. These dysregulated genes included genes related to canonical pathways, diseased and biological functioning, microglial, astrocytes, oligodendrocyte lineage cells, and the cell cycle [151]. These findings may help to identify genes and pathways involved in fetal ethanol-exposure-related diseases, such as FASD.

Ethanol exposure from P4–9 evokes gene expression changes in myelination-related genes, which are reduced after ethanol administration [152], and neuro-inflammation-related genes, which are increased after ethanol administration [153,154]. In line with elevated neuroinflammation, activated microglia were found in lobules V and IX of the cerebellum, possibly through alterations in CX3CL1-CX3CR1 signaling [153,154]. The increase in neuroinflammation could also be due to the increased chemokine MCP-1 expression after ethanol exposure, as administration of an MPC-1 synthase inhibitor or MPC-1 receptor antagonist reduces the percentage of activated microglia and decreases the expression of pro-inflammatory cytokines TNF-⍺ and IL-6 in the developing brain [155]. MCP-1 has been suggested to be crucial for ethanol-induced neurodegeneration. Caspase-3 levels, an apoptosis indicator, are increased after ethanol administration at P4 in the cerebellum [155,156] but are less elevated if ethanol is accompanied by an MPC-1 inhibitor and in MPC-1-deficient mice [155]. Similarly to MPC-1 inhibitors, nicotinamide has also the ability to diminish neurodegeneration after ethanol exposure. Ieraci and Herrera et al. (2018) found increased levels of activated Caspase-3 and PARP-1 as well as increased neurodegeneration in lobules III, VI, IX, and X after ethanol exposure at P4, with administration of nicotinamide reducing the ethanol induced neurodegeneration and apoptosis [156]. The specific PARP-1 inhibitor 3-ABA did not reduce Caspase-3 activation and neurodegeneration, suggesting that the working mechanisms of nicotinamide are mainly through reducing Caspase-3 activation. Other ethanol-induced effects on the cerebellum, such as neuroinflammation and degeneration, can be diminished by administration of a peroxisome-proliferation-activated receptor (PPAR)-*γ* agonist [137,153] or by low-intensity pulsed-ultrasound exposure [147]. Whether these protective mechanisms also apply in humans, and if this might be interesting for treatment options in cases of alcohol abuse during pregnancy, needs to be investigated.

Taken together, the literature is not consistent about the impact of maternal alcohol exposure on cerebellar maturation in humans and rodents (for a recent systematic review combining multiple species, see [157]). Rodent studies revealed that there is a clear negative impact of alcohol usage on cerebellar development but that the effects depend on distinct periods of vulnerability. The developing cerebellum seems to be most vulnerable to alcohol administration during the first week of the rodents’ postnatal life, which corresponds with the last trimester of pregnancy in humans. Within the cerebellum, and throughout the first postnatal week, alcohol sensitivity between PCs located in the different lobules varies. Whether such periods of vulnerability with differential gene expression profiles and different treatment options also exist in humans needs to be determined. For an overview of the above mentioned studies, see Table S1.

#### 5.1.2. Maternal Smoking—Impact on Cerebellar Maturation

Alterations in fetal brain maturation due to tobacco exposure have been observed in several human studies, including defective migration and maturation of PCs [105]. These developmental abnormalities suggest an association between maternal smoking and cerebellar malformations [158]. While some studies have not found significant differences in cerebellar volume [159], the broader body of research suggests that maternal smoking affects cerebellar function and structure in more subtle ways [160]. For instance, postmortem studies have revealed defective brain-derived neurotrophic factor (BDNF) expression in the cerebellar cortex [161], particularly in regions involved in respiratory control, further implicating tobacco exposure in impaired cerebellar development. Prenatally exposed children may also show altered brain activity, with greater recruitment of posterior brain regions, such as the cerebellum, possibly reflecting compensatory mechanisms for deficits in the prefrontal cortex [162,163].

Furthermore, cerebellar nicotinic receptors [105] have been implicated in the pathology of autism spectrum disorder [164,165,166,167]. These findings underscore the need for more research on the effects of nicotine, especially as newer nicotine products, such as e-cigarettes, gain popularity during pregnancy [168,169,170]. The long-term consequences of prenatal tobacco exposure on brain development remain a critical area of concern.

In rodents, a heterogeneous population of nicotinic acetylcholine receptors (nAChRs) have been demonstrated in GCs, the GL, the PCL, CN, and early during development (P0–P30) also on norepinephrin fibers [166,171,172,173], which suggests that nAChRs play a multitude of roles in cerebellar physiology. Activation of nAChRs elicits the release of GABA and norepinephrine in the developing and adult rat cerebellum [171,174,175]. Knocking out α7 nAChRs in a mouse model has a significant effect on the proteome of the cerebellum, particularly with regard to myelin sheath formation, ion transport, and glutamaterigc synapses [176]. α7 nAChRs are mainly expressed on PCs and already early during development. Between P3 and P5, there is moderate PC immunolabeling, which increases rapidly between P8 and P15, while at the same time, it disappears from rostral lobules. The receptor localization seems to follow a columnar organization in areas where it is located. Finally, at P20, α7 subunit labeling is found again in all PCs, although with lower intensity. This distinct temporal and spatial distribution suggests that α7 receptor expression is developmentally regulated, with a time course that parallels the final differentiation of PCs [177]. α7 receptors are less sensitive to nicotine compared to other nicotine receptors [178]. The development of primary cerebellar neuroblasts in the eGL is mediated by α3-nAChR subunits [179]. Thereby, nicotine could affect the general cerebellar development early during maturation, and, indeed, smoking is known to affect cerebellar development during different developmental stages.

Many studies have investigated the impact of smoking or nicotine exposure on the maturing brain. Nicotine, which is the psychoactive drug in tobacco, is able to cross the placenta, and, for long periods of time, it can even be found in higher concentrations in the fetus compared to the concentrations in the mother [180]. When the maturing embryo is exposed to nicotine, it has an inhibitory effect on the development of stem cells [181].

Prenatally, nicotine exposure during almost the whole pregnancy results in a significant increase in the density of dying PCs and a reduced density of surviving PCs in adolescent and adult rats [182,183]. Moreover, glial fibrillary acidic protein (GFAP) immunoreactivity expression is significantly increased in the GL and white matter of the cerebellum in adolescent and adult rats [182,183]. The vulnerability to maternal nicotine exposure is duration-sensitive, with more severe histomorphological PC differences after 2 weeks of daily prenatal nicotine exposure compared to 1 week [184]. However, one study states that nicotine administration for the three prenatal weeks does not alter PC numbers in the cerebellar vermis at P10 [185]. Nicotine exposure in the first postnatal week increases apoptosis in the GL [186], and nicotine exposure in the first and second postnatal week reduces PCs in the cerebellar vermis in rats [136].

Regarding nAChRs, maternal nicotine exposure during pregnancy leads do an upregulation of cerebellar α7 nAChRs in male rats [187]. Nicotine administration during the second and third postnatal week results in downregulation of cerebellar α7 nAChRs; although, administration limited to the first postnatal week has no significant effect on α7 nAChRs.

Although the evidence of nicotine-induced alterations in cerebellar development is not extensive, more research on other brain areas has pointed out the negative effects of pre- and postnatal nicotine exposure on cellular, molecular, and behavioral levels [188,189,190,191]. Furthermore, nicotine exposure in adult rats increases apoptosis in the white matter of the cerebellum [192], highlighting the importance of future research to unravel the effects of maternal nicotine exposure on the development of the cerebellum. For an overview of the above-mentioned cerebellar studies, see Table S2.

It is not only maternal nicotine exposure which can be harmful to development, but maternal smoking without nicotine can also negatively affect offspring. Studies show that maternal vaping without nicotine leads to neurological, behavioral, and epigenetic changes [193,194]. Regarding the cerebellum, maternal exposure to e-cigarettes containing propylene glycol and vegetable glycerol during the first three weeks of pregnancy in mice results in increased pro-inflammatory cytokine IL-6 levels in the cerebellum of adolescent offspring [195]. Increased levels of cytokine IL-6 indicate an increase in neuroinflammation. With the high rise in e-cigarettes popularity, and the stigma of being ‘safer’ compared to tobacco smoking, more research is important to underline the consequences and risks of maternal e-cigarette usage for different brain areas.

#### 5.1.3. Maternal Cannabinoid Usage—Impact on Cerebellar Maturation

Cannabis usage is the most commonly used illicit substance among pregnant women [196]. Δ^9^-tetrahydrocannabinol (THC), the main psychoactive compound, enters the fetus through the plasma with around 1/3 of the THC crossing the fetoplacental barrier [197], whereas, postnatally, it can enter the baby via breastmilk [198]. Prenatal cannabinoid (CB) exposure can cause preterm birth, leads to increase in admission in neonatal intensive care, and results in behavioral and social deficits [199,200,201,202]. All of the previous are known to affect healthy cerebellar development.

Regarding the direct effects of cannabinoids on the endogenous endocannabinoid system, the endogenous endocannabinoid system comprises endogenous endocannabinoids (eCB), the metabolic enzymes responsible for the formation and degradation of endocannabionids, and the cannabinoid receptors with interacting proteins. The endocannabinoid system exists from the earliest stage of pregnancy, in the preimplantated embryo and uterus, placenta, and in the developing fetal brain [203,204,205]. There are two types of cannabinoid receptors, type 1 (CB1R) and type 2 (CB2R). At gw14, CB1R can be found in the cerebellar cortex [203], and also, later in life, CB1R expression is widespread and high in the cerebellum of humans and rodents [206]. The majority of receptors are located on the presynaptic side of terminals received by PCs, and there is a moderate expression in the ML and low in the GL of the cerebellum [207]. CB1R are also located in mitochondria, where they modulate energy homeostasis [208]. 2-Arachidonoylglycerol (2-AG) is the most abundant eCB in the cerebellum, and diacylglycerol lipase α (DAGLα) is one of the major biosynthetic enzymes contributing to its production. Monoacylglycerol lipase (MAGL) is the key enzyme involved in 2-AG hydrolysis [209,210,211,212,213]. PCs play a key role in the production of 2-AG such that PCs are ready to activate CB1Rs in neurites approaching or traversing the PCL.

In humans, prenatal cannabis exposure has been associated with alterations in brain connectivity, affecting regions such as the dorsolateral, medial, and superior frontal areas, insula, anterior temporal lobe, posterior cingulate cortex, and cerebellum [214,215,216,217,218,219]. While some studies indicate that cannabis exposure during pregnancy may not lead to significant differences in cerebellar structure compared to controls, there is evidence that it can disrupt normal connectivity patterns [214,215,216,217,218,219,220]. For instance, increased connectivity between the hippocampus and cerebellum has been reported [221]. In addition, reduced connectivity between the caudate and cerebellar vermis, as well as hypo-connectivity between the anterior insula and cerebellum, has been observed in cannabis-exposed infants. This suggests that prenatal exposure to cannabis may impair the normal development of these networks. Functional MRI studies in young adults exposed to cannabis in utero show increased activity in the prefrontal cortex and decreased activity in the cerebellum, particularly during tasks involving response inhibition [222]. This altered activity may represent a compensatory mechanism, where increased prefrontal cortex recruitment compensates for cerebellar function deficits. These findings align with other studies that demonstrate increased activity in frontal regions and reduced cerebellar activity, highlighting how prenatal cannabis exposure can lead to long-term changes in brain function and connectivity [223].

Despite these findings, inconsistencies remain [196], with some studies reporting no significant differences in cerebellar volume or connectivity in cannabis-exposed individuals compared to controls. The variability in results may be due to differences in the timing and dosage of cannabis exposure, as well as the methods used to assess brain development [224]. These findings emphasize the need for further research, particularly in light of the increasing prevalence of cannabis use during pregnancy and its potential impact on fetal brain development [216].

In the mouse brain, CB1 receptors are expressed from E11 (5–6 weeks old human embryo) with gradually increasing levels of both mRNA and receptor density throughout the prenatal period. At E17.5–P3, CB1R is prominently expressed in long-range axons in the brainstem and cerebellum, and a role of eCB in the fasciculation of pontocerebellar, thalamocortical, and subcortical axons has been suggested [225,226,227]. By P5, CB1R expression becomes low in the long-range axons and prominent in neurites of radially migrating GCs and in the parallel fibers in the anterior and central zones. During the first two weeks postnatally, CB1R-expressing and -differentiating GCs are located in the anterior and central vermis, and paravermis but not in other cerebellar zones [225]. MAGL expression is high in PCs lining the primary fissure, likely to cause higher rates of 2-AG hydrolisis. 2-AG hydrolisis dampens eCB signaling through CB1R in differentiating GCs within lobes V–VI, likely revealing the specific eCB signaling within the anterior vermis [225].

CB knockouts show a reduction in the anterior cerebellar vermis size from the first postnatal week [196]. THC exposure from E5 to P20 leads to a reduction in glutamate transporter expressions of glial (GLAST) and neuronal (EAAC1) subtypes at P20, P30, and P70 [228]. One study shows that prenatal moderate levels of synthetic cannabinoid exposure from E3 until birth leads to no change in the CB1R levels and AMPA-GluA1, whereas NMDA-GluN2A is reduced. There is also a significant reduction in total MAO and certain markers of oxidative stress, which may link to the potential higher addiction chance in early adolescence [229,230,231]. Exposure to a low doses of cannabis for 5 min a day from E5.5–17.5 resulted in an increased cerebellar volume in mice at P60 [232]. Maternal cannabis exposure from E5 until birth also changes intrinsic membrane properties by increasing, for example, the firing rate of PCs through altering the membrane excitability by modulation of ion channels in P50 old rats [233]. The eCB system plays an important role in cerebellar development as well as its long-range connections with the MF and CF system and in PC cerebellar plasticity.

To summarize, cannabis abuse during vulnerable periods during pregnancy affects cerebellar development critically, leading to long-term effects.

#### 5.1.4. Maternal Opioid Usage—Impact on Cerebellar Maturation

The current “opioid crisis” affects populations across continents, and infants with prenatal opioid exposure are born almost every 15 min [234]. Incidences of opioid exposure are associated with an increase in the risk of perinatal problems, such as neonatal abstinence syndrome (NAS), prematurity, and low birth weight. Opioids are known to cross the placenta and the blood–brain barrier as well as the mother’s milk. Perinatal opioid exposure has a negative association with cognitive and motor outcomes persisting during school age [235].

High-affinity opioid-binding sites are associated with the ML in humans and high levels of opioid receptors have been found on human GCs, suggesting opioid-dependent maturation [236,237,238,239,240]. Volumetric studies revealed that newborns with prenatal opioid exposure exhibit reduced cerebellar white matter and/or smaller cerebellar volumes [241] compared to controls, with the changes persisting until adolescence [242,243,244,245]. However, in most studies, the impacts of covariates such as smoking and/or alcohol usage can hardly be measured because mothers in this group may often be users of multiple substances.

In mice, opioid receptors and peptides are widely expressed by developing cerebellar cells. Throughout development, the opioid peptides and receptor expression changes considerably (for review read [107]), indicating endogenous opioid-dependent maturation. Opioid receptors appear in peripheral tissues around E9.5 and in neural tissues around E11.5 [246]. Heroin and morphine activate μ-opioid receptors, δ-, and κ-receptors at high concentrations. During development, the eGL expresses μ-, δ-, and a putative ξ-receptor but no κ-receptors, whereas the adult cerebellum barely expresses opioid receptors, only low levels of δ-receptor [247,248,249,250].

In line with the fact that during the early stages of development, opioid peptides and receptors, such as μ-receptor, exist in the rodent cerebellum, different types of μ-drugs agonists lead to an inhibited growth of the cerebellum. The latter is most likely due to inhibition of GC neuroblast proliferation [247,251,252,253]. Methadone administration of mothers of dams during pregnancy and after birth leads to an attenuation of myelin development but an increased density and percentage of oligodendrocyte precursor cells and increased proliferative oligodendrocytes. Maternal methadone exposure furthermore leads to an increase in apoptosis of mature and myelinating oligodendrocytes at P7 in rats. Oligodendrocytes in the cerebellar white matter have been suggested to be more vulnerable to methadone than those in cerebral white matter, which is supported by the fact that the white matter of school aged children is only affected in the cerebellum but not cerebrum [254]. Opioids also affect the dendritic differentiation of GCs and PCs [255,256,257], furthermore supporting that throughout development, endogenous opioids support GC and PC proliferation, differentiation, and maturation; hence, after development, opioids fulfill a different or not such an important role anymore. Maternal opioid intake therefore may act through misleading the endogenous timing and sequence of endogenous neurotransmitter–receptor interactions, especially in eGL from E13 onwards in rodents.

### 5.2. Stress and Sleep and Cerebellar Development

#### 5.2.1. Perinatal Stress—Impact on Cerebellar Maturation

Changes in the cortisol levels of mammals can affect the neuronal development of the unborn offspring significantly. Indeed, in humans, severe maternal stress during pregnancy can influence fetal cerebellar development through a process known as ‘fetal programming’ [258,259]. This concept posits that variations in the intrauterine environment during critical fetal developmental phases can induce enduring changes in both the structure and function of the fetus, including the brain [260,261]. Such alterations arise when the fetus adapts or prepares for the expected postnatal environment based on these prenatal signals. Increased maternal anxiety can intensify the release of glucocorticoids and diminish the integrity of the placental barrier, facilitating greater glucocorticoid transmission to the fetus [262]. Additionally, heightened anxiety can reduce uterine blood flow, thereby negatively impacting fetal brain growth but not birth weight [261,262]. Such prenatal environmental changes may lead to cognitive, motor, and behavioral challenges in children [260]. Moreover, disruptions in neuroendocrine pathways like the hypothalamic–pituitary axis have been observed in offspring of mothers with heightened anxiety, suggesting potential long-term impacts on brain structure and function [259,263].

In a study by Buss et al. (2010), high pregnancy anxiety was associated with gray matter volume reductions in several brain regions, including the cerebellum [20], in offspring aged 6 to 9 years, as revealed by structural MRI scans [264]. Furthermore, prenatal exposure to maternal psychological stress is linked with increased sleep problems in toddlers and is associated with decreased fetal cerebellar–insular connectivity, although the specific mediating effects of fetal brain regions remain unidentified [264,265].

Similarly, in rats, stress can induce many cerebellar developmental abnormalities. For example, maternal stress at E7 and E14 results in decreased nuclear sizes of PCs and GCs, increased PC proliferation, increased density of PCs, reduced synapse-to-GC ratio, reduced GC-to-PC ratio, as well as decreased synaptophysin expression in the GCs during adolescence [266,267]. Likewise, maternal stress in mice during their third week of pregnancy results in long-lasting morphological differences in PCs located in the vermis of pups. The surface of the dendritic trees of the pups PCs increases during adolescence but decreases during adulthood, accompanied by an increase in anxiety-related behavior [268,269]. Prenatal stress induced by glucocorticoid administration at the end of the pregnancy results in reduced numbers of dendritic branches of the PCs and cerebellar weight in both adolescent and adult rats, increased levels of mGluR1 in adults [270], and irregularities in the eGL and PCL in juvenile rats [271].

Importantly, the negative impact of stress extends beyond pregnancy. For example, stress induced by daily corticosterone administration at P2–14 leads to a decrease in glucocorticoid receptor expression in the CN, while it impairs associative cerebellum-dependent learning later in life [272]. One hour of maternal separation between this same timeframe leads to decreased glucocorticoid receptors at P15 but increased glucocorticoid receptors later in life [273]. Along the same vein, stress induced by transportation of rodents during the second postnatal week leads to changes in the excitability of CN neurons [274].

Overall, the evidence for the negative effects of stress during pregnancy and early postnatal life on development of the cerebellum is robust, but the specific impact depends on the precise period of stress induction and presumably also on its intensity. For example, a relatively short period of 3 h of maternal deprivation a day during the first two postnatal weeks increases neurogenesis and cell density in the GL of rats, it increases the mRNA and protein levels related to neuronal growth and survival, and it increases the short-term myelinization [275,276]. On the other hand, 24 h of maternal deprivation during the second postnatal week increases cell death in the GL and eGL [277] and alters the number of astrocytes in the GL of rats [278]. These findings may be explained by differences in neuronal vulnerability to stress during the first weeks postnatally [279,280]. Indeed, during the first 2 weeks after birth, the stress response may be relatively mild, which comes with decreased corticosterone levels and reduced levels of the adrenocorticotropic hormone (ACTH) and corticosterone release after mild stressful events [279,280]. This ‘stress-hyporesponsive period’ is suggested to have a protective function for the maturing brain against elevated glucocorticoid levels [280]. However, during highly stressful events, the sensitivity to stress is increased, indicating that this stress-hyporesponsive period is probably only a mechanism to protect the brain against mild stressors, i.e., not life-threatening situations [281]. In line with this, the administration of high doses of glucocorticoids for only one day during these two postnatal weeks is associated with increased degeneration in the eGL, whereas low doses are not [282]. Moreover, long-term effects in the GL are still visible in adult mice following a glucocorticoid injection at P7; however, no differences in PC numbers in the PCL are identified [282]. It would be interesting to gain a better understanding of the beneficial impact of the stress-hyporesponsive period during early life on the cerebellum and if there might also be similar neonatal hyporesponsive periods regarding stress in humans (Table S3).

#### 5.2.2. Impact of Sleep Deprivation on Cerebellar Maturation

Considering that sleep deprivation is a major stressor of pregnant mothers, a study testing the effects of maternal sleep deprivation on fetal outcomes is of high importance [283]. Poor sleep quality is associated with adverse intrauterine physical growth and an increased risk of neurodevelopmental issues in children, affecting their cognitive abilities, behavioral development and learning abilities [284,285].

Accumulating evidence in rodent research shows that sleep deprivation during pregnancy affects the emotional and cognitive functions of offspring, with changes in hippocampal neurogenesis, hippocampus-dependent spatial learning and memory, and increased risk-taking behaviors in offspring [286,287,288,289,290]. The effects of maternal sleep deprivation on cerebellar development have not been documented.

Acute sleep deprivation in humans leads to a decreased level of attention and working memory as well as a reduced ability to respond to stimuli in a timely fashion [291]. A total of 36 h of sleep deprivation in men leads to changes in the functional connectivity of brain regions involved in psychomotor vigilance. It leads to increases in functional connectivity between subcortical areas and the cerebellum and to decreases between the cerebellum and cortical areas [292]. Whether acute maternal sleep deprivation during pregnancy affects cerebellar functionality and connectivity in mothers and fetuses similarly has not been studied yet. Hence, it seems overdue to study the effects of maternal sleep deprivation on intrauterine and fetal cerebellar development.

Furthermore, it is important to study how fetal sleep deprivation, especially during late gestation and early postnatal periods, affects cerebellar development [293,294]. Both during late gestational periods and early postnatal periods, newborn humans and rodent pups sleep almost 80% of their time. Moreover, it has been suggested that active sleep periods play a major role in the functional development of the cerebellum. For example, given that the cerebellum receives a copy of motor commands as well as subsequent signals about sensory feedback during sleep periods with muscle twitches, the cerebellum may be entrained during sleep to develop predictive coding of movements [295,296]. During such active sleep, synapses may be strengthened or weakened for the sensorimotor system to develop [297]. As a consequence, without sleep-related twitching, the cerebellum may not develop its distinct ability to process the motor commands and sensory feedback signals within the expected time period [298,299,300,301]. Therefore, it is not surprising that Tfap2b, which is a gene that acts during early embryonic stages, controls not only sleep in mice but also affects functioning of GABAergic neurons in the cerebellum [302,303]. In this regard too, it will be interesting to find out which sleep-control genes and how sleep restriction affects development of the cerebellum and/or that of other brain regions [304].

### 5.3. Intrauterine Growth Restriction—Impacts on Cerebellar Maturation

IUGR affects around 10% of human pregnancies and is associated with long-term motor and cognitive problems [305,306]. The cause for IUGR can be maternal factors such as undernutrition and maternal smoking but also placental and cord abnormalities, as well as fetal factors such as congenital heart disease. Since the causes for IUGR are heterogeneous, there is also overlap with the consequences described for the other insults in this review [307]. IUGR leads to a heterogeneous set of fetal clinical pathologies. Recent studies suggest that certain motor deficits in patients can result from abnormal cerebellar development due to IUGR [308,309].

In humans, instances of decreased cerebellar volume, or cerebellar hypoplasia, are frequently observed in fetuses experiencing preterm birth (i.e., babies born <37 weeks of gestation) and/or IUGR (also called fetal growth restriction: FGR) [310]. Alterations to typical cerebellar growth can occur because of influences that can be relatively direct or indirect [311]. The neuropathology underlying IUGR is intricate and unique compared to preterm infants without IUGR and term infants exposed to acute hypoxia. Research, spanning human imaging, post-mortem examinations, and animal models, often paints a picture of IUGR brains having diminished volume, compromised gray and white matter structures, and cellular anomalies. Specifically, gray matter regions exhibit fewer cells with a chaotic cortical configuration, whereas white matter appears immature with signs of inflammation and astrogliosis [312]. The structural connectivity, especially along motor and cortico-striatal-thalamic tracts, has been suggested to be altered in IUGR brains, correlating with adverse neurodevelopmental outcomes in affected children [312,313]. The risks of neurodevelopmental impairments in IUGR are modulated by the severity of growth restriction, its onset timing, the presence of relative “brain sparing”, and gestational age at birth.

Rodent studies have shown that bilateral uterine vessel ligation to restrict blood flow to the fetus can be used as a model for IUGR. Artery ligation in mice from E12.5 days onwards leads to cerebellar changes in myelination, especially when it is combined with hyperoxia [314]. Unfortunately, no specifics about the region of the cerebellum analyzed or the neuron types affected by demyelination were given. Applying artery ligation at E18 in rats towards the end of the first postnatal week leads to a 30% increase in the width of the eGL, while there is no difference in the width of the proliferative zone or the proliferating marker Ki67 in GCs. The increase in eGL following artery ligation may be partly because Bergman glia cells and their fiber density become disorganized and decreased [315,316]. Indeed, since the Bergman glia fibers normally guide the migration of the GCs from the eGL to the GL during early development, any structural disturbance in the Bergman fibers may affect GC transfer [315,316]. Additionally, the expression of genes that are necessary for a healthy migration to the GL may be affected following uterine vessel ligation, which could further worsen the deficits in cerebellar development. GC defects in turn may affect normal PC development, as suggested by a guinea pig study [317].

Maternal and postnatal malnutrition from E0–P21 of the rat increases lipoperoxidation and decreases superoxide dismutase [318], which may lead to lipid oxidative damage. Malnutrition of fetus during the last 5 embryonic days leads to reduced levels of glutamic acid decarboxylase (GAD) only at P2, examined with high-performance liquid chromatography (HPLC) of the whole cerebellum [319]. Compared to controls, HPLC also revealed an increase in the amino acids, alanine, and taurine. Thus, rodent data suggest that in babies with IUGR, the cerebellum is likely to be affected, since GCs cannot sufficiently migrate from eGL to GL, which in turn may impact development and myelination of PCs.

However, one must consider the limitations and caveats of the uterine vessel ligation animal model, since human placental insufficiency usually develops more gradually across the different gestational periods with the consequence that cerebellar development can be affected differentially with potential for compensatory mechanisms to be engaged.

### 5.4. Intrauterine Infections and the Impact on Cerebellar Maturation

Chorioamnionitis (CA) is an infection of the chorion and amnion of the mother, which can lead to a fetal inflammatory response, with adverse consequences for the developing fetal brain [320]. When brain inflammation is prolonged and/or becomes severe, it can exacerbate damage through further influx of cytokines, chemokines, and other inflammatory mediators released from glial cells. In humans, there is a strong causal link between CA, preterm brain injury, and the pathogenesis of severe postnatal neurological deficits, such as cerebral palsy [321]. When measured in premature infants, there is a significant association between exposure to CA and neurodevelopmental impairments from 18 to 30 months of corrected age [322,323,324], decreased cognitive performance at 5 years [321], and autism spectrum disorder [325]. The most frequent route that causes CA development in humans is the ascending microbial invasion from the lower genital tract. In a study conducted by Jain and colleagues [326], moderate to severe acute histological CA was found to elevate the risk of structural brain anomalies also in the cerebellum, as seen on MRI, both directly and by prompting premature birth.

In mice, one model to induce CA is ureaplasma-induced perinatal inflammation at E13.5, which induces a significant decrease in calbindin-positive neurons as well as a moderate decrease in myelin basic protein (MBP) [327]. PCs are the major calbindin-positive cell type. Another mouse model for CA makes use of inflammation-induced encephalopathy of prematurity driven by systemic administration of pro-inflammatory IL-1β. This model has been shown to interfere with the physiological roles of microglia in the cerebellum; indeed, inducing inflammatory activation with this approach results in perturbed oligodendrocyte development and myelination in whole cerebellum lysates used for Western blotting [328]. IL-1β administration during the first postnatal week, a timing equivalent to the last trimester for brain development in humans, leads to specific reductions in gray and white matter volumes of the mouse cerebellar lobules, most specifically lobules I and II, and the nucleus interpositus from the second postnatal week onwards. These volume changes, which can be detected with MRI as of the second week, are preceded by reduced proliferation of OLIG2+ cells as well as reduced levels of MBP and myelin-associated glycoprotein (MAG). Moreover, the density of IBA1+ cerebellar microglia is increased both during the first postnatal week and P45, with evidence for increased microglial proliferation during the first two postnatal weeks. CA also induces a significant enrichment of pro-inflammatory markers in microglia from the cerebellum and cerebrum, with the cerebellar microglia displaying a unique type I interferon-signaling dysregulation. In summary, perinatal inflammation driven by systemic IL-1β leads to cerebellar volume deficits, especially in lobules I and II but also other lobules, which likely reflect oligodendrocyte pathology downstream of microglial activation [328].

## 6. Vulnerable and Critical Periods in Cerebellar Development Affected by Intrauterine Insults

Lifestyle choices and environmental exposures of mothers can impact the cerebellar development of fetuses, potentially causing lifelong consequences for the structure and function of the cerebellum and leading to neurological motor and cognitive disabilities [2,20]. Abundant animal research has revealed the multifaceted negative effects of a hostile intrauterine environment on cerebellar development. Human studies in this area are limited, focusing often on postnatal cerebellar manifestations (e.g., neuroimaging measurements) while disregarding the impact of prenatal anomalies on individual cells, layers, different subareas of cerebellar development, and growth. Here, we provide a perspective on the potential critical periods for a healthy cerebellar development during gestation, highlighting opportunities to prevent risks for mothers and healthcare specialists of perinatal cerebellar brain injury considering rodent and human research.

In human and rodent studies on developmental disorders caused by insults, the cerebellum is still a relatively neglected brain region, and thus, the effects of insults on cerebellar development are underestimated and sometimes even controversial. It has, for example, been shown that stress during the second and third gestational period leads to changes in maternal care later in life as well as epigenetic variations [329], suggesting an effect on cerebellar functionality. What this explicitly means for cerebellar development still needs to be determined.

When the impact of insults on cerebellar development is analyzed, the focus lies often on PCs and GCs, since they form the sole output neurons of the cerebellar cortex and, most of all, neurons in the brain, respectively. We now know that depending on the time point, duration, and severity of the insult, different subareas, layers, and neuron types are affected. What makes the cerebellum different from other brain areas is that the critical periods are extended over the whole gestational period until 3–4 weeks postnatally in rodents and 3 years in humans. Considering cerebellar neurogenesis, proliferation, and migration, most likely, GABAergic interneuron proliferation is affected slightly later during gestation compared to excitatory CN neurons, but there are hardly any studies looking at the effects of an insults on all individual neuron types. This is surprising because CN neurons will be important to study since excitatory CNs develop first within the cerebellum, they are output neurons of the cerebellum, and they are the relay structure of the whole cerebellar cortex, being of major importance for cerebellar-thalamo-cortical communication [10,330,331,332,333].

When interpreting results of our review, it is important to consider four key factors. First, it must be mentioned that all information provided in this review is dependent on the study design chosen by the investigators. Therefore, it is inevitable that there are differences in cerebellar modifications, such as cell type, and timeframes when comparing the studies within and between different intrauterine and postnatal insults. This means that the information provided in this review must been seen as an overview; if different cell types are not mentioned to be affected by a specific intrauterine insult, this does not mean that they are unaffected. Additionally, in the cerebellum, a single neuron type could cause a chain reaction of malfunctioning cells due to the aforementioned developmental dependencies. 

Second, and as previously mentioned, there are differences in the development of the cerebellum as a whole, cerebellar subareas, and the molecular composition of cerebellar cell types comparing humans and rodents [40,41,42]. The folia complexity of human cerebellum is greater with enlarged hemispheres relative to the medial cerebellar vermis [22]. In humans, foliation and growth of the cerebellum takes place during gestation, whereas in rodents the largest growth is postnatal. Besides the surface area is greater and the neuronal subtype ratios differ significantly between individual cerebellar areas [53,334]. Next to that, the developmental timeline of individual cell types is different with slight regional differences between areas (Figure 1, Figure 2 and Figure 3 [9,23,57]). 

Third, the fact that there are regional differences in the vulnerability of the cerebellum to intrauterine and postnatal insults shows that all studies discussed in this review should be interpreted with care and that, where necessary, the lobules and, ideally, microzones should be reinvestigated and seen as individual functional regions. In most studies, it is not obvious whether researchers selected cerebellar areas to be analyzed a priori to the data gathering. One cannot see the cerebellum as one structure when it comes to vulnerability and functional development (see, e.g., [30]), even though it has a uniform appearance during adulthood.

Fourth, unlike human research [20], research in rodents permits identification of specific timepoints in cerebellar development that appear most critical for insults following manipulation. Brain development in rodent pups generally resembles the development of humans, in that the orders of their different gestational periods correspond relatively well [335], but extrapolating rodent studies to the human situation remains difficult as the absolute and relative durations of the different stages vary substantially [22,57,336]. As a result, critical periods of cerebellar development that are most vulnerable to specific agents vary among the different mammalian species. These differences must be considered when raising awareness and providing information for obstetricians and other healthcare professionals to eventually design strategies for preventing or rescuing related neurodevelopmental disorders. 

Below, we separately discuss the major mechanistic principles for the three prenatal trimesters in humans and discuss separately the first two postnatal weeks in rodents, where the development of the rodent cerebellum resembles the human cerebellum during the third gestational period. After, we discuss how different subareas of the cerebellum might be affected by intrauterine insults.

### 6.1. First Trimester (Until gw13 in Humans, Until E12/13 in Rodents)

Even though cerebellar development of mammals starts during the first trimester of pregnancy, hardly any study in humans or rodents focuses on the effects of intrauterine insults limited to this trimester. In humans, PCs are born within this period (beginning of the seventh gw, rodents between E10–E13.5) until the PC plate is formed (13th gw). Also, the eGL starts to develop (10–11th gw: humans, E12.5–16.5: rodents), and CN neurons.

The vulnerability of the cerebellum to drugs with abuse liability has been studied in humans and rodents [101,102,103,104,105,106,107]. However, human studies suffer from the fact that most studies rely on postmortem data or brain scans made after birth. Furthermore, most human mothers do not use excessive drugs only for a couple of days during pregnancy, but they continue throughout pregnancy, or they are polysubstance users. In rodents, maternal alcohol exposure for only one day at E8 or E9 leads to long-term effects on the cerebellar shape and short-term decreased volumes [123,124,125,126,127]. Alcohol exposure during the entire first gw leads to a decreased proportion of GABA receptors [128], having a high impact on PC neurogenesis. Maternal alcohol exposure from the first trimester onwards affects Wnt signaling, which has been shown to be important for cerebellar maturation and development [149]. eCB and opioid receptors are known to be expressed differently throughout development, and early heavy usage of cannabinoids and opioids will affect cerebellar development negatively. eCB and opioid receptors start being expressed around E11 (gw5–6) [225,226,227,246]. The eCB system plays an important role for cerebellar development and its long-range connections with the MF and CF [225,226,227]. Therefore, manipulating the eCB system through excessive usage of drugs will likely affect cerebellar development and the synaptic inputs through the MF and CF system [229,232]. Opioid receptors agonists during early pregnancy affect PC and GC proliferation negatively and can even lead to an attenuation of myelination. Maternal opioid intake can mislead the endogenous timing and sequence of neurotransmitter–receptor interactions, especially in the eGL from E13 rodents [250,251,252,253,254,255,256]. Maternal nicotine usage starting in the first week and ending in the last week of pregnancy has been shown to affect PC development [182,183]. Stress has also been shown to affect the nuclear size of PCs and GCs, as well as the dendritic structure of CN neurons, when it occurs one day during the first and one day during the second gw in rodents [266,267]. Studies on the effects of nicotine, sleep deprivation, IUGR, or CA solely during the first trimester on cerebellar development has not been published, which is a gap in cerebellar research.

To conclude, for most studies, the effects of insults on cerebellar development cannot be isolated and assigned to the first trimester. In rodents with genetic variations that affect the development of the cerebellar anlage, the mutation often leads to severe developmental problems, e.g., [74,79]. If insults are not deadly, developmental compensations, as is often seen in rodent research, might also occur, e.g., [337].

### 6.2. Second Trimester (gw13–26 in Humans, E13–Birth in Rodents)

During the second trimester of pregnancy, most cerebellar neuron types migrate. GCs differentiate from eGL and migrate to the GL along Bergman glia. UBCs and GABAergic interneurons develop and start migrating.

Excessive drug usage during the second trimester has also been shown to impact cerebellar development, especially if it occurs throughout pregnancy. In rodents, alcohol administration during a certain period of the second trimester is less harmful for PCs compared to other periods [130], although alcohol administration during the second trimester combined with the first and/or third trimester leads to changes in the GC, Bergmann glia, and PC development [131,132]. Effects have been shown to be regional [131]. Nicotine, cannabis, and opioid usage during the first and second trimester affects the cerebellum, specifically in terms of GC and PC survival and neuroinflammation [182,183,184,229,232,258,259,260]. Opioids, for example, are known to affect the dendritic differentiation of GCs and PCs [255,256,257], supporting that throughout development, endogenous opioids maintain GC and PC proliferation, differentiation, and maturation. Maternal opioid intake therefore may act through misleading the endogenous timing and sequence of endogenous neurotransmitter–receptor interactions, especially in eGL from E13 in rodents. Maternal stress between E14 and E21 affects the morphology of the PC dendrites in rodents [268,269,270]. IUGR and CA induced at E12.5 and E13.5 in rodents (end of first / beginning second trimester in humans), respectively, both lead to adverse effects on myelination and synaptogenesis [314,315,316,327,328]. CA furthermore has been shown to lead to a decrease in calbindin-positive neurons, which are PCs in the cerebellar cortex [327]. One must keep in mind that significant effects on the early development of one neuron type will most likely also affect the migration of the other cerebellar neurons [88,338]. Therefore, insults lasting longer, as often happens with CA, IUGR, as well as opioid-, nicotine-, or alcohol-addicted mothers, lead to adverse effects that might be rather general with yet important implications.

To summarize, insults affecting rodents’ embryos throughout the last weeks before being born affect mainly Bergmann glia, PC, and GC development by defects seen in the migration, morphology, myelinization, and synaptogenesis. To our current knowledge, no clear regional differences in the vulnerability of the cerebellum have been described when harmful insults occur exclusively during the second trimester. Hence, we know that, for example, opioid receptors are not equally distributed across the rodent and human cerebellum and thereby will lead to differential effects when opioid receptor agonists or antagonists are used.

### 6.3. Third Trimester (gw27–gw40 in Humans)

During the third trimester, the development of the cerebellum in humans is different from the development in rodents. In humans, the cerebellum grows and foliates, and neurons proliferate and migrate. Substance (ab)use, stress, IUGR, and CA in humans affect cerebellar development, but insults are often not specific for the third trimester but happen throughout pregnancy.

Recently, one study in non-human primates showed that CA during the third trimester leads to PCs loss and a disrupted maturation of GCs, with the PC loss being accompanied by decreased shh signaling from PCs to GC [339,340]. CA furthermore accelerated pre-oligodendrocyte maturation into myelinating oligodendrocytes, which is not in line with rodent research but in line with increased expression of MBP in the cerebellum of CA-exposed fetuses. These findings are also consistent with reported histopathological findings in individuals with autism and suggest a potential mechanism through which perinatal inflammation together with a change in the cerebellar excitation and inhibition balance through changes in myelination contributes to neurodevelopmental disorders in humans.

### 6.4. Two Weeks Postnatal Rodents

The development of the rodent cerebellum during the first two weeks after birth (P0–P14) resembles the cerebellum of a last trimester embryo in humans. Hence, this period of cerebellar development in rodents happens postnatally and is not reliant on maternal blood supply and the placenta anymore. Alcohol administration for only one day during the first postnatal week significantly alters cerebellar weight at P10 [101]. Throughout the first week, regional differences in the effects on volumes of the GL and ML as well as PC and GC loss have been shown. Ethanol exposure furthermore affects the CF to PC connections and microglia activation. Mainly, lobules I–IV and IX–X are affected by ethanol [101,134,136,137,138,139,141], which corresponds with human studies showing a decrease in volumes of lobules I–V of the vermis [341]. Lobule VII is less vulnerable during the beginning of the first postnatal week compared to the end. After the first postnatal week, the window of vulnerability for ethanol seems to close [133,135,141,142]. The CREB-gene is likely to play a role in protecting the cerebellum for ethanol-induced PC loss during this time period [134,143]. Nicotine and opioid usage during these first two postnatal weeks affect both the GCs and PCs [136,186,254,255,256,257]. It has been shown for methadone that it negatively influences myelination and that it leads to an increase in apoptosis of mature and myelinating oligodendrocytes at P7 in rat pups [255,256,257]. The eCB and CB1R are prominently expressed in long-range axons of the pontocerebellum [225,226,227] during the first postnatal weeks, and since eCB has been suggested to play a role in the fasciculation of pontocerebellar axons [225], usage of cannabis during that period or earlier can lead to detrimental effects on the timing and spatial distribution of postnatal MF spreading [342,343]. Since eCB also affect cerebellar PC plasticity significantly [210], a change in the timing and spatial distribution of eCB can thereby influence cerebellar development negatively. Also, stress leads to time- and dose-sensitive changes: mild stressors during the first and second postnatal week, also known as the ‘stress-hyporesponsive period’, induce GC proliferation, whereas more severe stress is related to degeneration in the GL [276,277,282]. In rodents, PCs seem less sensitive to stress during the first postnatal weeks [282]. Regarding alcohol, the vulnerability seems to be more time-specific: whereas administration during the first week after birth leads to both PC and GC loss, administration during the second week rarely affects PCs and GCs.

To conclude, in rodents, the first weeks postnatally are very vulnerable and lead to severe effects; however, dependent on the insults, there are certain critical periods where the cerebellum is more or less vulnerable. There are time periods in the rodents’ lives where the cerebellum is less vulnerable to both stress and alcohol. Whether there is also such a period in human cerebellar development is unknown. What has been shown is that, for example, language and working memory impairments vary in the degree according to the duration and amount of alcohol exposure, but the relation with cerebellar development is unstudied.

### 6.5. Differential Spatiotemporal Vulnerability of Cerebellar Development

A neglected area of research is to differentiate the effects of intrauterine insults on the highly heterogeneous individual subareas of the cerebellum (Figure 1). It has been shown that the anterior cerebellum, which connects most with the sensorimotor system matures before the posterior cerebellum, and, thus, early onset insults may lead to more severe or more general motor deficits compared to late onset insults. Due to receptor and eCB location, CB knock-out mice show a reduced size specifically in the anterior vermis of the cerebellum [225], also suggesting motor deficits. CB1R KO knock-out mice show impairments in fine motor forepaw coordination with no defects in gross motor coordination tasks, which is consistent with mild cerebellar ataxia [225]. Posterior cerebellar lesions may lead to more severe cognitive outcomes due to the connectivity of the posterior cerebellum with association cortices and because posterior cerebellar development follows that of the anterior cerebellum. Midline posterior vermal damage is more associated with behavioral dysregulations such as autism-like diseases. 

On another note, Wang and colleagues also mentioned that developmental diaschisis may evolve due to the deleterious effects of cerebellar lesions on the regions of the cerebral cortex to which the cerebellum projects [19,344,345,346,347,348,349]. In line with that, cerebellar output controls the pattern of ongoing activity in subcortical projection neurons, and aberrant development of those long-range connections, due to, for example, prenatal cannabis usage, can also lead to fine forepaw coordination impairments [217].

While the gross anatomy of the cerebellum looks similar across species and within the cerebellum of one species, the microscopic anatomy does not. Within the cerebellum, there is not only the classical functional subdivision into an anterior, posterior, and flocculonodular lobe but also into individual lobules, the vermis and the hemisphere, microzones, microcomplexes, and micromodules, all of which contain similar types of neurons that express distinct molecular markers with characteristic physiological properties [10,43,44,90]. Data suggest that distinct lobules and neuronal subtypes are affected differently, depending on the insult happening, which may at least partially be explained by a difference in the developmental timeline between areas [9,10,22]. The development of these molecular TOC profiles is tightly regulated, and the regional differences in genetic and thus molecular identity affect the intrinsic excitability as well the synaptic plasticity and eventually also the functionality of lobes, lobules, areas, microzones, microcomplexes, and micromodules as well as individual neuronal subtypes species specific. Ethanol leads to time-dependent and cerebellar-lobule-specific effects on PC development. As an example, while the literature is inconsistent about the vulnerability of lobule VI during the first postnatal week, PCs in lobule VII are only affected when exposed to ethanol at the end of this first week [135]. Moreover, lobules I–V and IX were more affected when exposed to ethanol at P4–5 compared to P6–7, suggesting that ethanol-vulnerability decreases over time and is cerebellar-region-specific. Together, these data suggest regional differences and time dependencies in the development of ethanol responsiveness in the cerebellum [135].

What also matters when it comes to regional differences is, whether the insult enters the cerebellum via, e.g., the blood vessels (Figure 1), the blood–brain barrier, or via breastmilk. Dependent on whether the insult affects the general cerebellum or only some local areas close to, e.g., a blood vessel, this will entail regional differences in vulnerability (Figure 1). As an example, IUGR is not only linked to oxidative stress and neuroinflammation in the fetus later in life, but it is also linked to placental insufficiency, which in turn can induce chronic fetal hypoxia [312]. Due to the distinct vesselation, hypoxia may affect cerebellar regions differently and lead to region-specific cerebellar oxidative stress, cytokine imbalance, redox dysregulation [350], region-specific deficits in neuronal organization, and/or axonal injury [309]. Oxidative stress and inflammation in the fetus are associated with impaired neurodevelopment and neurodevelopmental diseases such as autism spectrum disorder [351].

Thus, in addition to the timing and duration of the specific intrauterine insult to enter the cerebellum, the way of administration is also of importance when providing case-specific information for obstetricians and other healthcare professionals to eventually design strategies for preventing or rescuing related neurodevelopmental disorders.

## 7. Conclusions

In summary, the cerebellum is an early-developing brain region that undergoes rapid growth during gestation and continues to mature into late postnatal life in both humans and rodents. Despite its important role, it is often neglected in developmental research on intrauterine insults. When studied, cerebellar development is frequently treated as homogeneous, overlooking its complex and region-specific maturation. There is no single critical period applicable to all intrauterine insults; instead, each insult must be evaluated individually. For example, the development of opioid receptors creates an early gestational vulnerability in cerebellar development, whereas ethanol exposure has a more severe impact during the third trimester or postnatally. Therefore, the critical periods of vulnerability in both humans and rodents depend on the timing, duration, location, and severity of each specific insult. Recognizing these nuances is essential for understanding and mitigating the effects of intrauterine challenges on cerebellar development.

## Data Availability

Not applicable.

## Supplementary Materials

The following supporting information can be downloaded at https://www.mdpi.com/article/10.3390/cells13221911/s1, Table S1: Overview table summarizing literature on timepoints, exposure methods and effects of prenatal and early postnatal alcohol exposure on cerebellar outcome measures in rodents; Table S2: Overview table summarizing literature on timepoints, exposure methods and effects of prenatal and early postnatal nicotine exposure on cerebellar outcome measures in rodents; Table S3: Overview table summarizing literature on timepoints, exposure methods and effects of prenatal and early postnatal stress exposure on cerebellar outcome measures in rodents.
