# Supplementary material for: Impact of Intrauterine Insults on Fetal and Postnatal Cerebellar Development in Humans and Rodents
Source: Cells. 2024 Nov 19;13(22):1911. doi: 10.3390/cells13221911 (PMC11592629; doi:10.3390/cells13221911)
Supplement: Supplementary file 1 [file cells-13-01911-s001.zip › cells-3290296-supplementary.pdf]

Table S1. Overview table summarizing literature on timepoints, exposure methods and effects of prenatal and early postnatal alcohol exposure on cerebellar outcome measures in rodents

**Time-point alcohol**	**Ethanol/day**	**Exposure methods**	**Rodent**	**Effects cerebellum** (compared to controls, unless mentioned differently)	**Reference**
20 days pre-gestation + E0-birth + P0-P21	20% ethanol in water	Oral	Rats	Decreased cerebellar weight (at P21 and P60, not at P30).<br>Thicker eGL (at P21).<br>Longer GC dendrites and bigger somas (at P60, not at P21 or P30).<br>Morphological differences of Bergmann glia cells.	González-Burgos & Alejandre-Gómez, 2005 [132]
E0-E8	~10.6 g/kg	Oral	Mice	Decreased GABAa receptor subunit α-1 proportion in cerebellum (at P80).	Nguyen et al. 2020 [128]
E5-birth	~6 g/kg	Oral	Rats	Increased ROS and lipid peroxide levels (at week 6).<br>Increased MAO activity (at week 6).<br>Decreased expression of GLUR1, PSD95 and ILK, and increased expression of Caspase-3. No differences GSK3 β expression (at week 6).<br>No differences in Complex V and VI activity (at week 6).	Bhattacharya et al. 2018 [129]
E6-birth	18% or 37% of daily caloric content	Isocaloric liquid diet	Rats	3 of the 84 Wnt pathway related genes were downregulated (ethanol: 37%, at P10)<br>33 of 84 Wnt pathway related genes were downregulated (ethanol: 37%, at P35)<br>Increased expression of Fzd4, Fzd6, Axin2, Dixdc, EP300 (at P3) and Wnt5b (at P20).<br>Decreased expression of Fzd4, EP300 (at P10) and Pzd4, Fzd6, Wnt5a, EP300 (at P20).<br>Decreased expression of β-catenin, Cyclin D1, c-Myc (at P3, not at P10 and P20).	Gundogan et al. 2013 [149]
E6-birth	24% of caloric content	Isocaloric liquid diets	Rats	Increased insulin and IGF-1 receptor levels (at P30).<br>Decreased IRS-1 protein, Akt, and GSK-3β levels (at P30).<br>Decreased relative levels tyrosine phosphorylated insulin and IGF-1 receptors (at P30).<br>Decreased expression of Wnt5a, Fed 6, Didxc, and Axis 2 (at P20).<br>Decreased levels of Jagged 1, HES-1, AAH, HIF-1α, but not of Notch 1 and FIH (at P30).	Tong et al. 2013 [150]
E7	2x 2.9 g/kg	Intraperitoneal injection dam	Mice	No effect on volume cerebellum (at E17)	Godin et al. 2010 [127]
E8	2x 2.8 g/kg	Intraperitoneal injection dam	Mice	Shape differences in cerebellum, no volume differences (P100 (Fish et al. 2018), and at P45 (Fish et al. 2016))	Fish et al. 2016; 2018 [125,126]

E8	2x 2.8 g/kg	Intraperitoneal injection dam	Mice	Decrease cerebellar volume (at E17).	Parnell et al. 2009 [123]
E9	2x 2.9 g/kg	Intraperitoneal injection dam	Mice	Decrease cerebellar volume (controls at E16.5, experimental group at E17)<br>Shape differences of cerebellum (mainly inferior, and mainly at the right side)	Parnell et al. 2013 [124]
E13-E18	5 g/kg	Oral intubation dam	Rats	No effect in number of PCs (at P10).<br>No effect on cerebellum-bodyweight ratio (at P10).	Marcussen at al. 1994 [130]
E12-19 + P2-9	Chamber levels between 3-8 g/dl (increasing over age)	Oral (vaper)	Mice	Decreased numbers of inhibitory interneurons ML and GL of in lobule II (at P16).<br>Lower numbers of PCs in lobules II, IV-V and IX (at P16).<br>Decreases volumes of lobules II, IV-V, VI-VII, IX and X of the vermis (at P16).	Nirgudkar et al 2016 [131]
P2-4	4.4 g/kg	Intraperitoneal injection	Mice	Decreased numbers of PCs and GCs (at P25).<br>Area loss of GL and ML (at P25).	Karaçay et al., 2008 [134]
P3-5	3.5 g/kg	Gavage	Mice	Decreased number of PCs (P6, lobule IX).<br>Decreased number of microglial and altered morphology (P6, lobule IX).<br>After ethanol and PPAR-γ agonist: increased number of microglial cells and PCs compared to ethanol condition (at P6, lobule IX).	Kane et al. 2011 [137]
	8.03 g/dL	Vaper inhalation	Rats	Decreased number of PCs in the vermis (at P6 all lobules, and at P45 lobules I-III and IX-X, not IV-VII. Not at P4).<br>Decreased thickness eGL (at P6, but not P4 orP45). No differences in thickness inner GL (at P6).<br>Increased IL-1β (after 20h withdraw at P4 and P6) and TNF-α (after 20h withdrawal at P4). No differences in TGF β and IL-10 expression.<br>Increased overall expression TGF β.<br>Morphology differences microglia associated with microglia activation (at P6, lobules IV-VII and IX-X).<br>Increased GFAP expression eGL, PCL, inner GL (at P6, all lobules).	Topper et al. 2015 [139]
P4	6.6 g/kg	Gastrostomy tube	Rats	Decreased volume cerebellum (at P10).<br>Decreased PCs of lobules IV- IX and X (not lobules VI and VII). No effect with 3.3 g/kg ethanol (at P10).	Goodlett et al. 1989 [101]

	5 g/kg	Subcutaneous injection	Mice	Increased activated Caspase-3 (8 hours after ethanol administration)) and PARP-1 (8 and 12 hours after ethanol administration) levels.<br>Decreased Caspase-3 levels when ethanol administration was followed by 1g/kg nicotinamide, but not by 3-ABA (0 or 2 hours after ethanol administration).<br>Increased neurodegeneration measured by FJB+ cells in lobules III, IV, IX and X (12 hours after ethanol administration). Decreased neurodegeneration and PARP-1 activation when ethanol administration was followed by nicotinamide, but not by 3-ABA.	Ieraci and Herrera 2018 [156]
	2x 2.5 g/kg	Subcutaneous injection	Mice	Increased expression MCP-1 (8 hours after first injection, at P4).<br>Increased expression TNF-α and IL-6. Decreased expression TNF-α and IL-6 after Bindarit (MCP-1 synthesis inhibitor) or RS504393 (CCR2 antagonist) administration compared to only ethanol administration (8 hours after first injection, at P4).<br>Increased expression cleaved Caspase-3. Decreased expression cleaved Capase-3 after Bindarit (MCP-1 synthese inhibitor) or RS504393 administration compared to only ethanol administration (8 hours after first injection, at P4).<br>Increased percentage activated microglia (in sections brain, cerebellum). Decreased percentage activated microglia after Bindarit (MCP-1 synthese inhibitor) or RS504393 administration compared to only ethanol administration (8 hours after first injection, at P4).	Zhang et al. 2018 [155]
	6.6 g/kg	Gastrostomy tube	Rats	Decreased number of PCs (at P27).	Goodlett and Eilers 1997 [133]
	4.4 g/kg	Intraperitoneal injection	Mice	Increased mRNA expression of CREB compared to ethanol administration at P10 and controls (at P4).<br>Reduced Cyclin D2 expression compared to ethanol administration at P10 (at P4).<br>Decreased BDNF expression (at P4).	Karaçay et al. 2008 [134]
	4.5 g/kg	Intragastric intubation	Rats	Decreased numbers of PCs (Lobule I, at P14).<br>Decreased intensity and volume of stained CF terminals / PC volume (Lobule I, ML and PCL, at P14).<br>Decreased intensity of colocalization of stained CF terminals and stained PCs / PC volume (Lobule I, ML and PCL, at P14).<br>Non-significant differences with 3.6 g/kg/day ethanol.	Pierce et al. 2010 [141]
	4.5 g/kg	Intragastric intubation	Rats	Decreased number of PCs (Lobule I, at P40).<br>Decreased volume of CF terminals / PC volume (Lobule I, ML, at P40).<br>Decreased intensity (in ML) and volume (in ML and PCL) of colocalization of stained CF terminals and stained PCs / PC volume (Lobule I, at P40).<br>Decreased colocalized CF puncta and PC / PC (Lobule I, ML, at P40).	Pierce et al. 2011 [142]

	4 g/kg	Intragastric gavage	Mice	Dysregulation of 2440 genes related to canonical pathways and diseased and biological functions (see paper for specific categories and genes) (at P5).<br>Dysregulation of 175 genes associated with microglia (at P5).<br>Upregulation of microglia genes associated with neurodegenerative diseases (at P5).<br>Dysregulation of 58 genes associated with astrocytes. These genes were associated with acute injury and pan-injury (at P5).<br>Dysregulation of genes associated with oligodendrocyte lineage: 65 oligodendrocyte precursor cells, 14 committed oligodendrocyte precursor cells, 45 myelin forming oligodendrocytes, and 1 mature oligodendrocyte (at P5).<br>Increase genes associated with positive regulation of G2-M phase transition, and increase genes associated with negative regulation of G1-S and G2-M phase transition (at P5).	Holloway et al. 2023 [151]
P4-5	6.6 g/kg	Gastrostomy tube	Rats	Decreased weight cerebellum and cerebellum-body weight ratio (at P21).<br>Decrease in number of PCs in lobules I-VI, VIII-X of the vermis (at P21).	Hamre and West 1993 [135]
	4 g/kg	Intragatric gavage	Mice	Dysregulation of 1348 genes related to canonical pathways and diseased and biological functions (see paper for specific categories and genes) (at P6).<br>Dysregulation of 105 genes associated with microglia (at P6).<br>Upregulation of microglia genes associated with neurodegenerative diseases (at P6).<br>Dysregulation of 33 genes associated with astrocytes. These genes were associated with acute injury, pan-injury, and chronic neurodegenerative diseases (at P6).<br>Dysregulation of genes associated with oligodendrocyte lineage: 32 oligodendrocyte precursor cells, 3 committed oligodendrocyte precursor cells and 24 myelin forming oligodendrocytes (at P6).<br>Increase genes associated with positive regulation of G1-S phase transition, and increase genes associated with negative regulation of G1-S and G2-M phase transition (at P6).	Holloway et al. 2023 [151]
P4-6	4.5 g/kg	Intragastric intubation	Rats	No differences in firing frequency PCs, higher asymmetry index of inter spike intervals (Lobule I, P13-15).<br>Increased IPSCs frequency and increased amplitude of Ih current (PCs, Lobule I, P13-15).<br>Decreased numbers of basket and stellate cells, and decreased number of PCs (Lobule I, at P23-25).<br>Increased number of basket and stellate cells per PC, and increased number of basket cells per PC (Lobule I, at P23-25).<br>Increased volume of fluorescence voxels of HCN1 per PC (Lobule I,<br>P41-48).	Light et al. 2015 [140]

	4.5 g/kg	Intragastric intubation	Rats	Decreased numbers of PCs (Lobule I, at P14).<br>Decreased intensity and volume of stained CF terminals / PC volume (Lobule I, ML, at P14).<br>Decreased intensity of colocalization of stained CF terminals and stained PCs / PC volume (Lobule I, ML, at P14).<br>Non-significant differences with 3.6 g/kg/day ethanol.	Pierce et al. 2010 [141]
	4.5 g/kg	Intragastric intubation	Rats	Decreased number of PCs (Lobule I, at P40).<br>Decreased volume of CF terminals / PC volume (Lobule I, ML, at P40).<br>Decreased intensity (in ML) and volume (in ML and PCL) of colocalization of stained CF terminals and stained PCs / PC volume (Lobule I, at P40).<br>Decreased colocalization of CF puncta and PC / PC (Lobule I, ML, at P40)<br>Decreased distance from ML-PCL border to surface (at P40).	Pierce et al. 2011 [142]
P4-9	4 g/kg	Intragastric gavage	Mice	Increased mRNA expression IL-1β, TNF-α (at P10 and P15, not P45), CCL2 and IL-1α (P10).<br>Morphology differences microglia (associated with activated microglia, at P10 lobule V).<br>No changes in mRNA expression of anti-inflammatory cytokines IL-1ra, IL-33, TGF-β1, TGF-βR type 1 and type 2 (at P10). No changes in mRNA expression NLRP1α, NLRP1β, NLRP3 and ASC (at P10).<br>Decreased mRNA expression IL-23 subunit p19 and IL-12Rβ1, but no changes in IL-12 subunit p35 and IL-12/IL-23 subunit p40 expression (at P10).<br>Decreased expression of CX3CL1, but not CX3CR1 (at P10).<br>Increased gene expression of NGP and NOX-2 (at P10).<br>Decreased RAG1, NOS1, CD59a, S1RP5, PTPN22, GPR37 and Serpinb1b (at P10).	Kane et al. 2021 [154]
	4 g/kg	Gavage	Mice	Increased mRNA expression of IL-1β, TNF-α and CCL2 (at P10).<br>Increased cell area and decreased cell territory microglia (lobule V cerebellar cortex, at P10).<br>After ethanol and PPAR-γ agonist: Decreased IL-1β and TNF-α expression, no differences in CCL2 expression. Reduced cell area and increased cell territory microglia (at P10).	Drew et al. 2015 [153]
	4 g/kg	Intragastric gavage	Mice	Reduced expression of NG2, Bcas1, CNPase, ENPP2, ENPP6, MBP, MOBP, MOG, PLP1 (at P10).<br>Reduced gene expression of Olig1, NKX6.2 and MYRF (at P10).<br>Reduced gene expression of GPR17, Opalin, and Tspan2 (at P10).<br>Reduced gene expression of MAG, OMG, FGF1 (at P10).<br>Reduced gene expression of Connexin 47, Claudin 11, TPPP, Hrh3, and Transferrin (at P10).	Niedzwiedz-Massey et al. 2021 [152]
	2.2 mg/g	Intraperitoneal injection	Mice	Decreased numbers of GCs and PCs, decreased PC density, and decreased GC volume (at P10).	Todd et al. 2022 [143]

				In mice with CREB knock-out in PC after ethanol administration: Decreased numbers of PCs and PC volume (compared to CREB knock-out controls and compared to CREB-positive ethanol exposure mice (at P10). Reduced rotarod performance (at P40). No difference in GCs (at P10).	
	5.25 g/kg	Gastric intubation	Rats	Decreased volumes lobules I-IV and IX-X (at P42).<br>Increased density of microglia in lobules I-IV and IX-X (at P42).	Gursky et al. 2020 [144]
	2.5 g/kg	Gastrostomy tube	Rats	Decreased numbers of PCs in lobules I-IV and IX-X vermis (at P10)<br>Decreased cerebellum-body weight ratio (at P10)	Marcussen at al. 1994 [130]
	4 g/kg	Gastrostomy tube	Rats	Decrease weight cerebellum (at P9).<br>Decrease PCs vermis (at P9).	Chen et al. 1998 [136]
	5 g/kg	Subcutaneous injection	Mice	Lobules IV/V vermis:<br>Increased IL-1β (female and male) and CCL2 (male) expressionF (at P10).<br>No differences in microglia density, spacing index or morphology in lobule IV/V (at P60, fixed tissue).<br>No differences in motility index and surveillance ratio (at P60, *in vivo* two photon imaging). Increased motility index in PCL compared to ML in females, and males+females both exposure to ethanol. Increased surveillance ration in PCL compared to ML in males+females, but not separately.<br>No differences in microglia-PC interactions in ML. Some effects in microglia-PC interactions in PCL in females (at P60, fixed tissue).<br>Decreased PC linear frequency (=amount of PC/length of PCL).<br>No differences in microglia volume in all layers (P60, fixed tissue).<br>Decreased PC volume fraction in PCL females (at P60, fixed tissue).<br>No differences in microglia-PC interactions in PCL and ML (at P60, *in vivo* two photon imaging).	Cealie et al. 2024 [145]

	5 g/kg	Subcutaneous injection	Mice	In vivo two photon imaging in lobules IV/V:<br>No effects on microglia motility- and surveillance index in ML or PCL (at P25-31).<br>Increased motility index in PCL microglia compared to ML microglia in males. Increased surveillance index in PCL microglia compared to ML microglia in males, females and combined (at P25-31).<br>No effects morphology microglia in ML or PCL when sexes combined. (at P25-31)<br>No effects in microglia-PC interactions in PCL (at P25-31).<br>Some non-significant effects in microglia-PC interactions in ML of females (increased interactions of whole microglia with whole PCs, increased microglia processes with non-branch areas of PC dendrites, increased microglia somas with non-branch areas of PC dendrites). No effect in microglia-PC interactions in ML in branched areas of PCs dendrites (at P25-31).	Cealie et al. 2023 [146]
P5	6 g/kg	Gastrostomy tube	Rats	Decreased number of PCs in lobule I of the vermis, but not at 4.5 g/kg or 2.5 g/kg ethanol (at P6).<br>Decreased cerebellum-body weight ratio, but not at 4.5 g/kg or 2.5 g/kg ethanol (at P6).<br>Increased neurodegeneration PCs between 12-18 hours after treatment (at P6).	Lee et al. 2008 [138]
P5-6	6.6 g/kg	Gastrostomy tube	Rats	Decreased weight cerebellum and cerebellum-body weight ratio (at P21).<br>Decrease in number of PCs in lobules I-VI, VIII-X of the vermis (at P21).	Hamre and West 1993 [135]
P6-7	6.6 g/kg	Gastrostomy Tube	Rats	Decreased weight cerebellum and cerebellum-body weight ratio (at P21).<br>Decrease in number of PCs in lobules I-X of the vermis (at P21).	Hamre and West 1993 [135]
P7-8	6.6 g/kg	Gastrostomy tube	Rats	No effect on cerebellar weight or number of PCs in vermis (at P21).	Hamre and West 1993 [135]
P7-9	4.5 g/kg	Intragastric intubation	Rats	No effect on number of PCs at (Lobule I, at P14).<br>Increased colocalized volume (ML and PCL) and intensity (PCL) of stained CF terminals and stained PCs / PC volume (Lobule I, ML, at P14).<br>Non-significant differences with 3.6 g/kg/day ethanol.	Pierce et al. 2010 [141]
	4.5 g/kg	Intragastric intubation	Rats	No effect on number of PCs at (Lobule I, at P40).<br>Decreased colocalized volume of staining CF terminals and staining PCs / PC volume (Lobule I, ML, at P40).	Pierce et al. 2011 [142]
P8-9	6.6 g/kg	Gastrostomy Tube	Rats	No effect on cerebellar weight or number of PCs in vermis (at P21).	Hamre and West 1993 [135]

P8-10	4.4 g/kg	Intraperitoneal injection	Mice	No effect on numbers of PCs or GCs (at P25).<br>No area loss of GL or ML (at P25).	Karaçay et al., 2008 [134]
P9	6.6 g/kg	Gastrostomy tube	Rats	No effect on number PCs (at P27).	Goodlett and Eilers 1997 [133]
P9-10	6.6 g/kg	Gastrostomy Tube	Rats	No effect on cerebellar weight or number of PCs in vermis (at P21).	Hamre and West 1993 [135]
P10	4.4 g/kg	Intraperitoneal injection	Mice	Less increased mRNA expression of CREB compared to ethanol administration at P4 and controls (at P10).<br>Increased Cyclin D2 expression compared to ethanol administration at P4 (at P10).<br>Decreased BDNF expression (at P10).	Karaçay et al. 2008 [134]
P10-12	5 g/kg	Intraperitoneal injection	Rats	Reduced numbers of GCs in inner GL and ML, and thinner eGL (age not specified).<br>After ethanol and low-intensity pulsed ultrasound: Increased numbers of GCs in inner GL and ML.	Bolbanabad et al. 2017 [147]
P12-13	6.6 g/kg	Gastrostomy tube	Rats	No effect on cerebellar weight or number of PCs in vermis (at P21).	Hamre and West 1993 [135]

* The studies are sorted in ascending order of exposure timepoints. E = Embryonic day; P = Postnatal day; PC = Purkinje cell; GC = Granule cell; PCL = Purkinje cell layer; GL = Granule cell layer; eGL = external granule cell layer; CF = Climbing fiber; ML = Molecular layer; GABA = Gamma-aminobutyric acid; ROS = Reactive oxygen species; GFAP = Glial fibrillary acidic protein, MAO = Monoamine oxidase; IPSC = Inhibitory postsynaptic potentials; Ih = Hyperpolarization activated inward current.

Table S2: Overview table summarizing literature on timepoints, exposure methods and effects of prenatal and early postnatal nicotine exposure on cerebellar outcome measures in rodents.

**Timepoint nicotine**	**Nicotine / day**	**Exposure methods**	**Rodent**	**Effects in cerebellum** (compared to controls, unless mentioned differently)	**Reference**
E0-E21	15mg/ 21 days (~0.7mg/ day)<br>Or<br>25mg/ 21 days (~1.2mg/ day)	Subcutaneous pellet	Rats	For both 15mg and 25mg nicotine pellets:<br>No effect on weight of cerebellar vermis (at P10).<br>No effect on numbers and density PC in vermis (at P10).	Chen et al. 2003 [185]
E4-20	3.3 mg/kg in bacteriostatic water	Subcutaneous implantation osmotic pump dam	Rats	Increased density dying PCs (at P30 and P60), and decreased density of surviving PCs (at P30).<br>Increased GFAP immunoreactivity in GL and white matter (at P30 and P60).<br>NB: Control animals were also treated with bacteriostatic water.	Abdel-Rahman et al. 2005 [182]

E4-20	3.3 mg/kg in bacteriostatic water	Subcutaneous implantation osmotic pump dam	Rats	Increased acetylcholinesterase activity in female cerebellum, not male (at P90).<br>Decreased density surviving PCs in female offspring (at P90).<br>Increased GFAP in GL and white matter in female offspring (at P90).<br>NB: Control animals were also treated with bacteriostatic water.	Abou-Donia et al. 2006 [183]
E4-21	6 mg/kg in bacteriostatic water	Subcutaneous implantation osmotic pump dam	Rats	Increased α7 nAChR binding in males (at P4 and P7).	Slotkin et al. 2004 [187]
E5-12	2x 0.8 mg nicotine + 10 mg tar and 10 mg CO	Smoking machine	Mice	Note: this study did not do a statistical examination.<br>Little damage PL - shrinkage soma and cytoplasm PC (at E13).	Al-Amri et al. 2021 [184]
E5-19	2x 0.8 mg nicotine + 10 mg tar and 10 mg CO	Smoking machine	Mice	Note: this study did not do a statistical examination.<br>Increased damage PC - PC shrinkage soma and damage cytoplasmic structures (at E20).	Al-Amri et al. 2021 [184]
P1-7	3x 2mg/kg	Oral nicotine liquid	Rats	Increased apoptosis in inner GL of both anterior and posterior lobules (at P8).<br>No effect on morphology neurons in inner GL, eGL and PC (at P8).	Huang et al. 2007 [186]
P1-4	2x 0.3 mg/kg or 2x 3 mg/kg	Subcutaneous injection	Rats	No significant effect α7 nAChR binding (at P5).	Slotkin et al. 2004 [187]
P4-9	6mg/kg	Gastrostomy tube	Rats	Decreased number of PCs in vermis (at P9).	Chen et al. 1998 [136]
P11-14	2x 0.3 mg/kg or 2x 3 mg/kg	Subcutaneous injection	Rats	Decreased α7 nAChR binding (at P15).	Slotkin et al. 2004 [187]
P21-24	2x 0.3 mg/kg or 2x 3 mg/kg	Subcutaneous injection	Rats	Decreased α7 nAChR binding (at P25).	Slotkin et al. 2004 [187]
Adult (age 3 months) Duration exposure: 57 days	2x 4.8mg	Chamber with airflow	Rats	Increased apoptosis in white mater (at ~5 months).	Wallauer et al. 2018 [192]

* The studies are sorted in ascending order of exposure time points. E = Embryonic day; P = Postnatal day; PC = Purkinje cell; GC = Granule cell; PCL = Purkinje cell layer; GL = Granule cell layer; eGL = external granule cell layer, CO = Carbon monoxide.

Table S3. Overview table summarizing literature on timepoints, exposure methods and effects of prenatal and early postnatal stress exposure on cerebellar outcome measures in rodents.

**Timepoint stress**	**Stress exposure methods**	**Rodent**	**Effects in cerebellum** (compared to controls, unless mentioned differently)	**Reference**
E7+E14	6 hours a day of restraining dam in close-fitting wire mesh cylinder	Rats	Decreased volume proportion GC nuclei to GL (at P30).<br>Decreased diameter GC nucleus (at P30).<br>Decreased density of synapses and number of synapses per neuron (at P30).	Ulupinar & Yucel 2005 [266]
E7+E14	6 hours a day of restraining dam in close-fitting wire mesh cylinder	Rats	Decreased diameter PC nucleus (at P30).<br>Increased density and numbers of PCs (at P30).<br>Decreased diameter GC nucleus (at P30).<br>Decreased GC to PC ratio (at P30).<br>Increased percentage BrdU+ PC (proliferation) (at P30).	Ulupinar et al. 2006 [267]
E14-20	3x 45 minutes a day restraining dam in a close-fitting plastic tube	Mice	Increased dendritic area per PC at P22 and P52 but decreased at P82.<br>No effect in elevated plus maze (at P22, P52 and P82).	Pascual et al. 2010 [268]
E14-21	3x 45 minutes a day restraining dam in a close-fitting plexiglas tube	Mice	Decreased dendritic area PC (at P82, but not at P52).<br>Reduced time spend in central zone of elevated plus maze, indicating anxiety (at P82, not at P52).	Pascual et al. 2015 [269]
E17-19	Intraperitoneal injection 0.2 ug/g body weight corticosterone + 2.5% ethanol and 5% propylene glycol	Rats	Decreased thickness of eGL and decreased cell density in eGL (at P12).<br>Decreased nuclear and AgNOR area of GC progenitor in the eGL (at P12).<br>Increased nuclear area of PC, no differences in PC density (at P12).	Rivas-Manzano et al. 2021 [271]
E20	2x subcutaneous betamethasone phosphate injection of 170 μg/kg	Rats	Decreased number dendritic branches of PCs (at P22, P52 and P82).<br>Increased mGluR1 expression (at P52 and P82, not at P22).<br>Decreased weight cerebellum (at P22, P52 and P82).	Pascual et al. 2017 [270]
P1	Injection of 3.0mg/kg dexamethasone	Mice	No difference degeneration eGL (4h after injection, at P1).	Noguchi et al. 2008 [282]

P1-14	Maternal separation 3 hours a day	Rats	Increased corticosterone levels (at P60, not at P15). Increased BrdU+ cell density in folia GL (at P15 and P60), and fissure (at P60). Increases cell density GC in both folia and fissures (at P15 and P60).	Roque et al. 2019 [276]
P2-14	Daily corticosterone injection of 1mg/kg dissolved in sesame oil	Rats	Decreased glucocorticoid receptor expression in posterior interpositus nucleus (at week 14-18). Decreased conditioned response amplitude day 6, 8, 9 and 10 in eye blink conditioning.	Wilber et al. 2011 [272]
P2-14	Maternal separation 1 hour a day	Rats	Decreased expression glucocorticoid receptor in posterior interpositus nucleus male at P15 but increased at P21 and at adult.	Wilber et al. 2009 [273]
P4	Injection of 3.0mg/kg dexamethasone	Mice	Increased degeneration eGL (4h after injection, at P4).	Noguchi et al. 2008 [282]
P7	Injection of 3.0mg/kg dexamethasone	Mice	Increase Caspase-3+ cells/apoptosis in eGL (4, 6 and 8h after injection, (at P7)). Increased degeneration in eGL after dexamethasone doses of 0.1; 0.3 and 3.0 mg/kg, not at 0.03; 0.01; 0.003 or 0.0 mg/kg (4h after injection at P7). Reduced GCs in inner GL, no effect on PCs in PCL (at week 7). Reduced Caspase-3 activation and apoptosis in Puma-deficient and Bax, Bak-deficient mice compared to wild type after dexamethasone injection, but not in p53-deficient mice (at P7).	Noguchi et al. 2008 [282]
P7-8	Transportation rodent	Rats	Increased transient bursting in lateral nucleus compared to animals transported at P13 (at P14).	Schneider et al. 2013 [274]
P9-10	Maternal deprivation for 24 hours	Rats	No difference in Fluoro-Jade C positive cells (in inner GL of vermis, at P13). Increased GFAP positive cells per mm^3 in inner GL and white matter (males, at P13). Decreased GFAP positive cells per mm^3 in inner GL (females, at P13).	Llorente et al. 2009 [278]
P10	Injection of 3.0mg/kg dexamethasone	Mice	Increased degeneration eGL (4h after injection, at P10).	Noguchi et al. 2008 [282]
P10-15	Maternal separation 3 hours a day	Rats	Increased mRNA and protein levels of BDNF, OMgp and TrkB (at P16, not at P30). No differences in NGF, TrkA, p75 NTR or NgR levels (at P16 and P30).	Miki et al. 2014 [275]
P12	Maternal deprivation for 24 hours (no food and water)	Rats	Increased ApopTag labelled cells/cell death in GL and eGL (at P13).	Zhang et al. 2002 [277]

P13	Transportation rodent	Rats	Decreased rebound index distribution compared to animals transported at P7-8 (at P14). No differences in input resistance.<br>Increased levels of corticosterone and decreased weight when transported at P13 compared to P7-8 (at P14).	Schneider et al. 2013 [274]
P14	Injection of 3.0mg/kg dexamethasone	Mice	No difference degeneration eGL (4h after injection, at P14).	Noguchi et al. 2008 [282]

* The studies are sorted in ascending order of exposure timepoints. E = Embryonic day; P = Postnatal day; PC = Purkinje cell; GC = Granule cell; PCL = Purkinje cell layer; GL = Granule cell layer; eGL = external granule cell layer; h=Hour.
